# Cardiac myofibril networks induce shear stress

**DOI:** 10.1038/s41540-026-00696-1

**Published:** 2026-04-02

**Authors:** L. A. Murray, A. P. Quinn, C. Pinali, D. J. Collins, V. Rajagopal

**Affiliations:** 1 Department of Biomedical Engineering, Faculty of Engineering and IT, The University of Melbourne, Melbourne, VIC Australia; 2 Division of Cardiovascular Sciences, Faculty of Biology Medicine and Health, University of Manchester, Manchester, England; 3 Graeme Clarke Institute, University of Melbourne, Parkville, VIC Australia; 4 Baker Department of Cardiometabolic Health, Melbourne Medical School, The University of Melbourne, Melbourne, VIC Australia; 5 Australian Research Council Centre of Excellence in Mathematical Analysis of Cell Systems (MACSYS), The University of Melbourne, Melbourne, VIC Australia

**Keywords:** Biophysics, Cardiology, Computational biology and bioinformatics, Engineering, Physiology

## Abstract

Myofibril arrangement is critical to cardiac muscle function in health and disease. Historically, analysis of the impact of myofibril organisation on force and cell contraction has relied on the assumption of uniaxial arrays. However, improvements in imaging indicate that myofibrils form complex networks, though how these networks modulate force has yet to be explored. Here, morphological analysis of sheep left-ventricular cardiomyocytes is utilised to inform a non-linear finite element model of cell contraction. Analysis of deep learning segmentations of z-discs demonstrate that myofibrils are oriented about the contraction axis (mean $=0.03^{\circ}$) but deviate locally by up to $30^{\circ}$ (standard deviation $=6.56^{\circ}$). Simulations produce unique deformations for geometries informed by myofibril orientations, displaying internal rotation and off-axis deformations. Moreover, anisotropy generates shear stresses distinct from the uniaxial case, demonstrating spatial relationships that balance shear across the cell and a correlation between shear stress and z-disc orientation. These findings highlight the impact of myofibril networks on forces during cell contraction.

## Introduction

Cardiac muscle cells produce the forces underpinning each heartbeat. These forces are a product of synchronous cell-shortening, facilitated by large structures called myofibrils^1,2^. Myofibrils span the rod-like myocytes longitudinally in arrays, serially connecting 1.8–2 $\mu {\rm{m}}$ long contractile protein assemblies, sarcomeres, which shorten up to 20% during activation^2,3^. Force is transmitted throughout the myofibril by z-discs which connect adjacent protein filaments, provide structural stability, and attach the myofibril to the cell membrane. Myofibril force production is facilitated by an influx of Ca^2+^ which enables interactions between actin and myosin filaments, producing shortening and decreasing the distance between adjacent z-discs^2,4^.

However, shortening dynamics are non-ideal. How sarcomeres behave during contraction has been demonstrated as non-uniform^5,6^. Experiments have indicated that during contraction some sarcomeres show no initial change, or even lengthen^5^, with pre-stretch increasing behavioural homogeneity^5,6^ in what may facilitate the Frank-Starling relationship^7–9^ at a cellular level. Further, the alignment of neighbouring sarcomeres, and thus, insurance of in-phase beating, is regulated by the local mechanical environment^10–12^. Changes to mitochondrial-sarcomere contact during development modulates the expression of aligned sarcomeres^10^. These modulations may be due to strain^11^, as researchers have found a correlation between the environmental elasticity and sarcomere alignment^12^, which could help improve understanding of sarcomere assembly^13^. Nevertheless, while the fundamental mechanisms of force generation have long been understood, there is increasing appreciation of the complexities and physiological implications resulting from the intracellular directions along which these forces are generated^14,15^.

Myofibril orientation has been historically presumed as uniaxial about the cell’s longitudinal axis^1,16,17^. These assumptions relied upon limited transmission electron microscopy (TEM) imaging, facilitating investigation in sarcomere structure and theories of contraction, but providing minimal 3D spatial context on global myofibril arrangement^16,18^. Therefore, intracellular stress has been historically decomposed into an *active* (axial) component produced by myofibrils, and *passive* (off-axial) components attributed mostly to the cytoskeleton^19–21^. This assumption has been perpetuated in subsequent biophysical models^22–27^ and experimental studies^20,28–30^, though deeper examinations of myofibrils, particularly in mammalian samples, may provide a more detailed understanding of intracellular stresses and their relevance to cardiac physiology and disease conditions.

Importantly, volumetric EM imaging has demonstrated that myofibrils may display splits^31,32^, are asynchronously aligned^33,34^, and form interconnected branches^15,35^. Goldspink^17^ originally highlighted myofibril structure in mice bicep brachii, observing that distal sarcomeres ‘split’ into smaller ‘daughter’ sarcomeres along the myofibril length, later suggesting splitting acts as a modulator of tension development or proliferation in fast- and slow-twitch muscles^32,36^. These splitting events were also observed by Tomanek^37^ in kitten skeletal muscle. More recently, Jayasinghe et al.^33^ applied laser scanning confocal miscopy to study z-discs as an indicator of myofibril structure, observing that myofibrils are helictical over myocyte length. The images of rat, rabbit, and human cardiomyocytes similarly display an asynchronous alignment of z-discs, purporting that these structures may positively influence contraction synchrony. Further, with focused ion beam-scanning electron microscopy, Willingham et al.^15^ highlighted that myofibril networks form in fast-, slow-twitch, and cardiac myocytes through sarcomere branching events. The evolutionary conservation of these branching events was then demonstrated by Ajayi et al.^35^ through selective gene-knockout of *Drosophila* myocytes, both groups hypothesising an impact on myocyte force production. Nevertheless, how such multiaxially aligned myofibrils impact intracellular stress has not been investigated.

An understanding of stress dynamics within the cell is critical for appreciating function in health, disease, and exercise. Cardiac myocytes respond to their mechanical environment, modulating membrane channels, calcium signalling, and force transmission^38–40^. These regulatory behaviours may help myocytes respond to altered loading conditions in disease states^41^, as well as facilitate development^10,42^, and accommodate demand^22^. Describing intracellular stresses is key to understanding the mechanosensitive behaviours of key organelles such as mitochondria, which have been shown to deform during contraction^43^ and modulate calcium levels^44^, and the nucleus, whose migration is impacted via myofibril force^42^. Still, while experimental designs incorporate off-axis stress behaviour^45,46^, virtually all biophysical models assume myofibrils produce zero active shear stress.

**Fig. 1:**
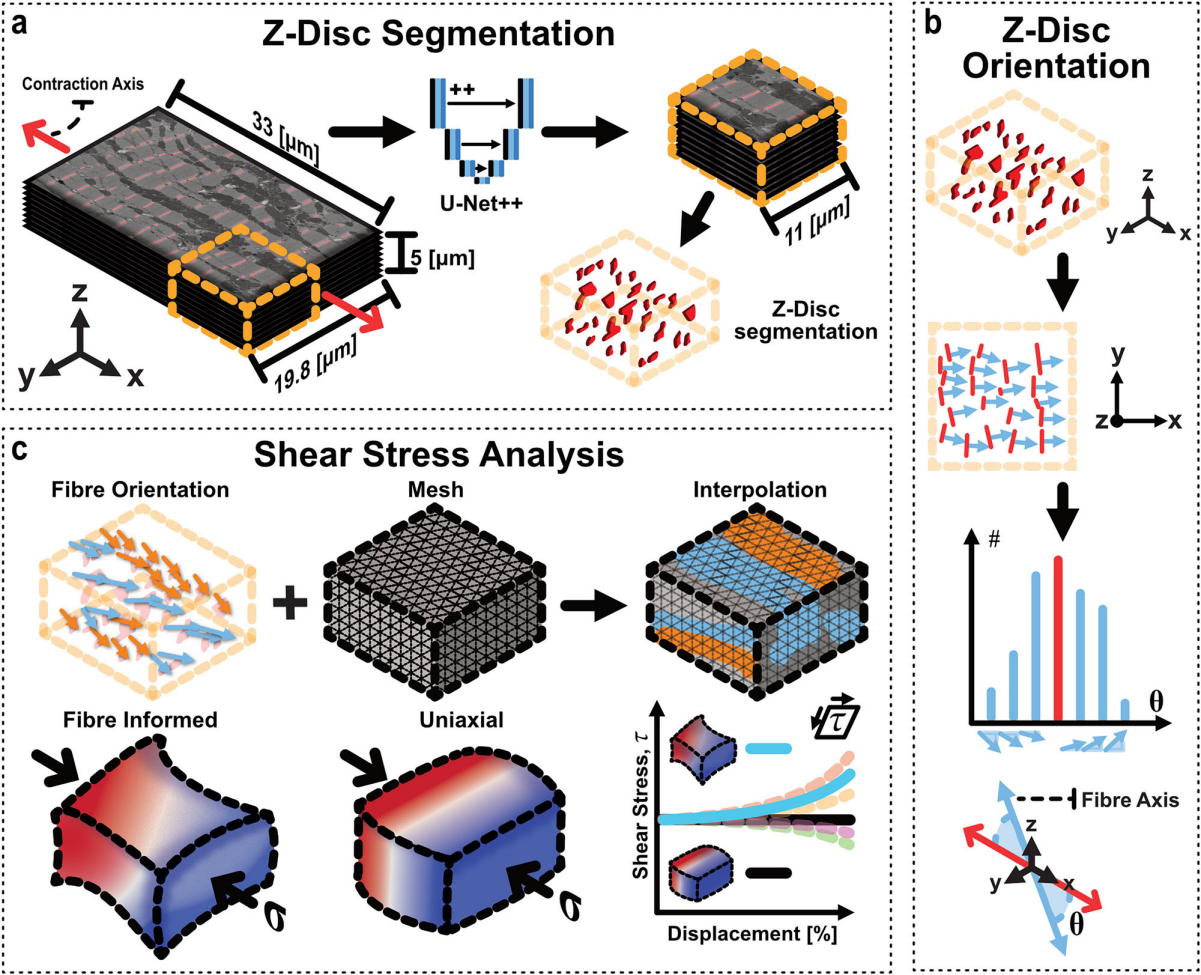
Visual abstract demonstrating the workflow from cardiomyocyte segmentation through to morphological analysis and mechanical simulation. **a** Z-Discs (red in EM image) are isolated from portion of sheep left-ventricular cardiomyocyte (33 × 19.8 × 15 μm; one 5 μm volume indicated here) through the U-Net++ Machine Learning model instance segmentation. **b** Isolated segmentations of z-disc are analysed with principal component analysis to quantify orientation of fibre in reference to contraction axis of cell. **c** Fibre orientation interpolated into non-linear mechanical model for contraction simulation. Fibre-informed simulations and uniaxial tests are contracted and shear stress quantified. Quantification indicates that shear stress in fibre-informed cases produce a distribution not reproduced by uniaxial model.

In this work, we examine the intracellular stresses in a striated cardiomyocyte resulting from off-axis z-disc orientations. This process is highlighted visually in Fig. 1. Here, serial-block face scanning electron microscopy (SBF-SEM) images of sheep left ventricular myocytes were segmented to create a physiologically informed biophysical model. A U-Net++^47^ was trained on manual segmentations to facilitate morphological analysis of z-discs as a proxy for measuring myofibril orientations. This analysis indicates that myofibrils permute at a distribution of angles within the network about the contraction axis (Fig. 1, red arrow; $\mu =0.03^\circ$, $\sigma =6.56^\circ$). Angles were then interpolated into a hyper-elastic finite element (FE) model using the FEniCSx^48–51^ open-source package. The FE model is parameterised to fit existing experimental trends for myocyte tension^20,28,52–54^. Integration of myofibril multiaxial alignment results in shear behaviours that are not reproducible with a uniaxial assumption. Further, a correlation between shear stresses and orientation is found with spatial distributions of shear transitioning across the cell width. Taken together, this study indicates that incorporating multiaxially alignment in biophysical models is vital for describing intracellular stresses in cardiac myocytes.

## Results

### Z-disc segmentation reveals myofibrils are not uniaxially aligned

**Fig. 2:**
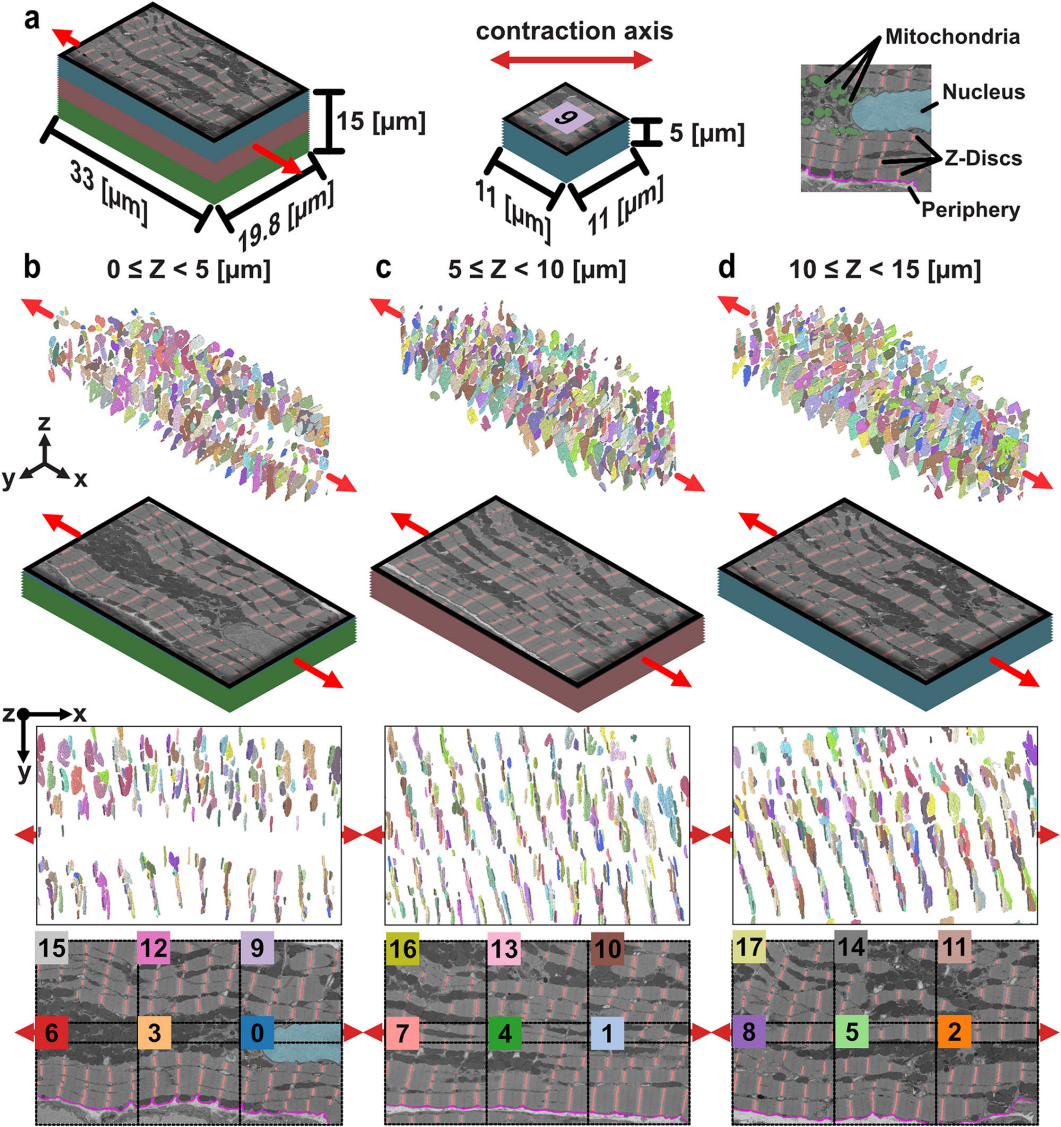
Z-disc segmentation from cardiomyocyte volume and split into test regions. **a**, (left) Key indicating dimensions of full cell segment ($33\times 19.8\times 15\,\mathrm{\mu m}$), broken into three stacks (z-discs in red). (middle) Dimensions of test regions ($11\times 11\times 5\,\mathrm{\mu m}$) displayed. (right) Extraction from Region 0 with key cell landmarks indicated (mitochondria in green, nucleus in light blue, z-discs in red, and periphery in pink). Visualisation of z-disc instance segmentations, electron microscopy (EM) images, and test regions for **b** $0\le z < 5\,\mathrm{\mu m}$, **c** $5\le z < 10\,\mathrm{\mu m}$, and **d** 1$0\le z < 15\,\mathrm{\mu m}$. **b**–**d** Row 1 (top), Orthogonal render of z-disc segmentations with contraction axis indicated. Row 2, orthogonal view of EM slices. Row 3, 2D top-down view of z-disc segmentations aligned left-to-right with contraction axis. Row 4 (bottom), 2D top-down view of EM slices (nucleus in light blue, z-discs in red, and periphery in pink) with overlay of numbers of regions for identification; dimensions as indicated in (**a**).

Isolated z-discs create varied patterns that do not predominantly align with the contraction axis. The larger 3D cell volume is partitioned into smaller volumes and regions (Fig. 2a, green, red, and blue). The bottom layer (Fig. 2b) is characterised by a large longitudinal grouping of mitochondria that span between edges, interrupted only by a nucleus on the proximal end. The boundaries of this mitochondria region and the nucleus contort the neighbouring sarcomeres’ orientation and shape, similar to results shown elsewhere^55^. This structure is more pronounced in the top-down 2D render shown at the bottom of Fig. 2b, where the z-disc axial alignment is clearly impacted by the mitochondria and the nucleus.

In each volume, the regular $\sim 1.9\,\mathrm{\mu m}$ spacing of z-discs along myofibrils is apparent (see Fig. 2b–d bottom row), although z-discs are not vertically aligned over width (see Supplementary Fig. 1). Asymmetry in alignment between myofibrils has recently been explored^33^, however the z-discs in this sample only exhibit alignment across width within myofibrils bundles, with a loss of this coherence between myofibrils that are spatially separated. Z-discs do not appear aligned over the full cell width.

Differences in myofibrillar arrangement patterns are apparent near the cell’s lateral periphery and nuclei. 2D visualisation of the slices (Fig. 2b–d bottom) highlight the z-discs at the cell periphery (all slices) and about the nuclei (Fig. 2b bottom). The cell periphery aligns myofibrils with the cell contraction axis, with a reduction of orientation changes particularly apparent in the second volume (Fig. 2c). Investigation into z-discs surrounding the nuclei, however, show a contouring of bodies about its border (Fig. 2b bottom). In both cases, statistical testing did not indicate any significant differences in orientations about these cell landmarks (see Supplementary Figs. 2–4).

### Myofibrils vary as much as ${\boldsymbol{30}}^{\boldsymbol{\circ }}$ from longitudinal axis

**Fig. 3:**
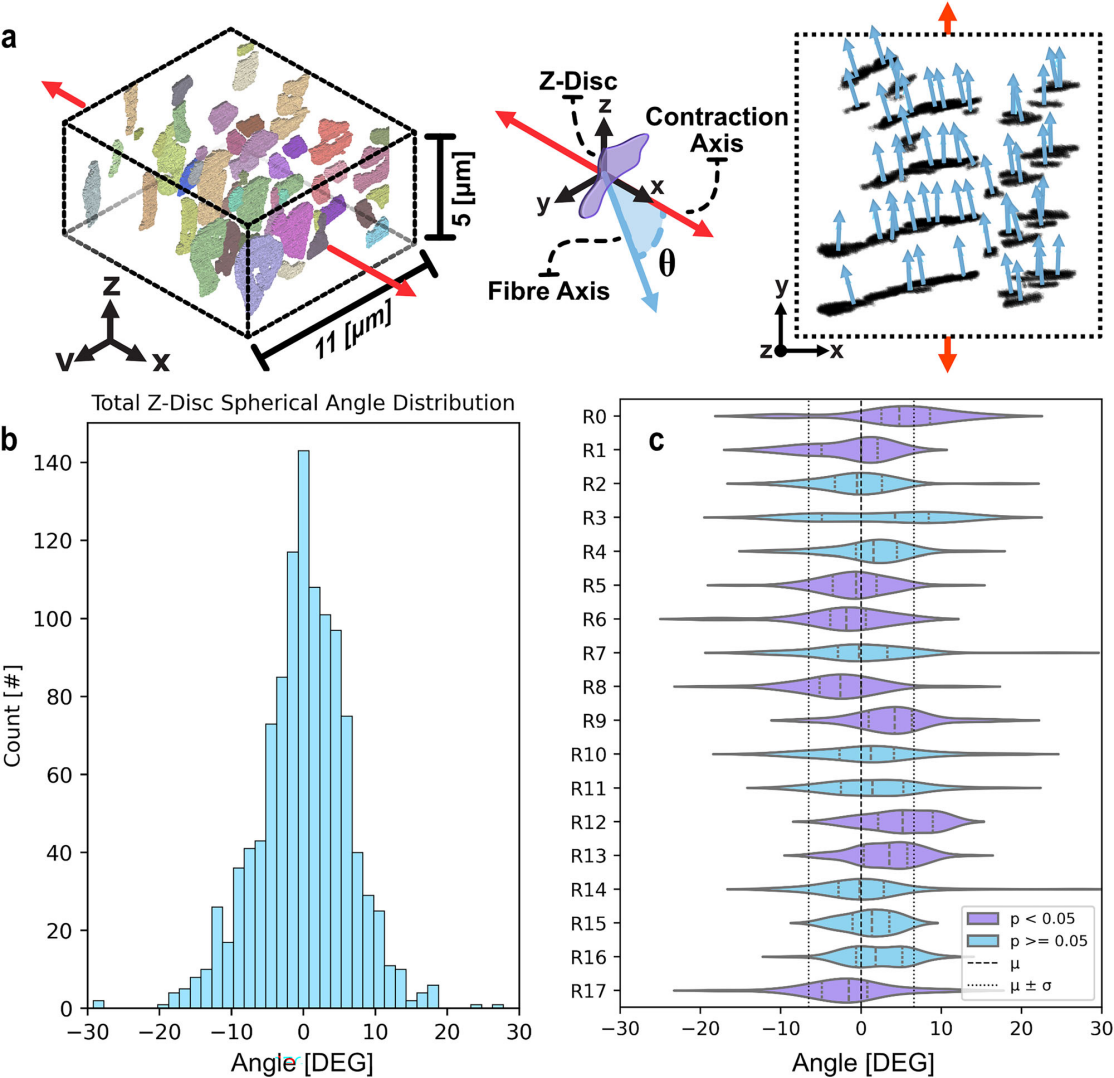
Isolation of spherical angle distributions from principal components analysis (PCA) of segmentation data. **a** (left) Region 7 rendered in orthogonal view. (middle) Diagrammatic indication of relationship between z-disc, fibre-axis, and contraction axis; the spherical angle is computed through the vector angle between the z-disc normal vector and contraction axis. (right) 2D top-down view of PCA computed normal vectors to segmentations from Region 7 shown top-left. **b** Histogram of global orientation values for all segmented z-discs, bin size: $1.5^\circ$. **c** Violin plots with quartiles shown for orientation data per test region. Purple regions indicate statistical significance between regional orientation values and global set. Significance was determined with Mann-Witney U test and Welch’s t-test $p < 0.05$. Vertical dash (--) and dotted (:) lines indicating global quartiles.

Principal component analysis (PCA) of segmented z-discs indicates that myofibrils throughout the cell are oriented between $-30^\circ$ and $30^\circ$ of the contraction axis (Fig. 3; standard deviation $\sigma =$ $6.56^\circ$; mean $\mu =$ $0.03^\circ$), which was calculated from the cell’s long axis (see Methods). This distribution occupies a similar range to Feinberg et al.’s measurements upon cardiomyocytes^56^, however the ‘normal’ shape here is more in line with expectations. To compute this angle the isolated z-discs were processed to determine the *normal* vector (Fig. 3a) through the third principal component. The z-disc is directly connected to the *actin-* and *titin-*filaments which run perpendicular to the z-disc body and parallel with contraction^1^. The normal vector is then compared to the contractile axis of the cell to determine spherical deviation (see Fig. 3a middle illustration).

Myofibrils are oriented symmetrically about the major axis ($n=1138$, ${skew}=-0.16$) with a standard deviation ($\sigma$) of $6.56^\circ$ and mean ($\mu$) of $0.03^\circ$ (see Fig. 2b). The large kurtosis value ($\kappa =3.49$) of this distribution indicates a concentration of values within the first standard deviation. Indeed, whilst a uniaxial model of myofibrils presumes complete alignment, only $\sim 12 \%$ of instances had an orientation within $1^\circ$ of the major longitudinal axis. The distribution was validated against another cardiomyocyte; a similar orientation profile was produced (see Supplementary Fig. 5 and Supplementary Table 2).

Regional orientations exhibit significant deviations in distribution and mean with respect to the main axis. Compared to the global set with Mann-Whitney U test and Welch’s t-test, $9$ of the $18$ test sets demonstrated significance (see Fig. 3c; see Supplementary Fig. 2). Whilst region size impacts statistical relevance, these distributions nevertheless highlight the regional variability in myofibril arrangement. Further, of regions exhibiting a statistically significant orientation distribution, all but one (R17) were immediately adjacent to another significant region. Of the $10$ significant regions, $5$ included the cell periphery and 2 included the nucleus, though these landmarks did not significantly impact adjacent orientations when tested independently with the same Mann-Whitney U test and Welch’s t-tests (see Supplementary Fig. 3). Broadly, each region demonstrates strong contraction axis alignment with orientation profiles like the global cell (see Supplementary Table 3 and Supplementary Fig. 6).

**Fig. 4:**
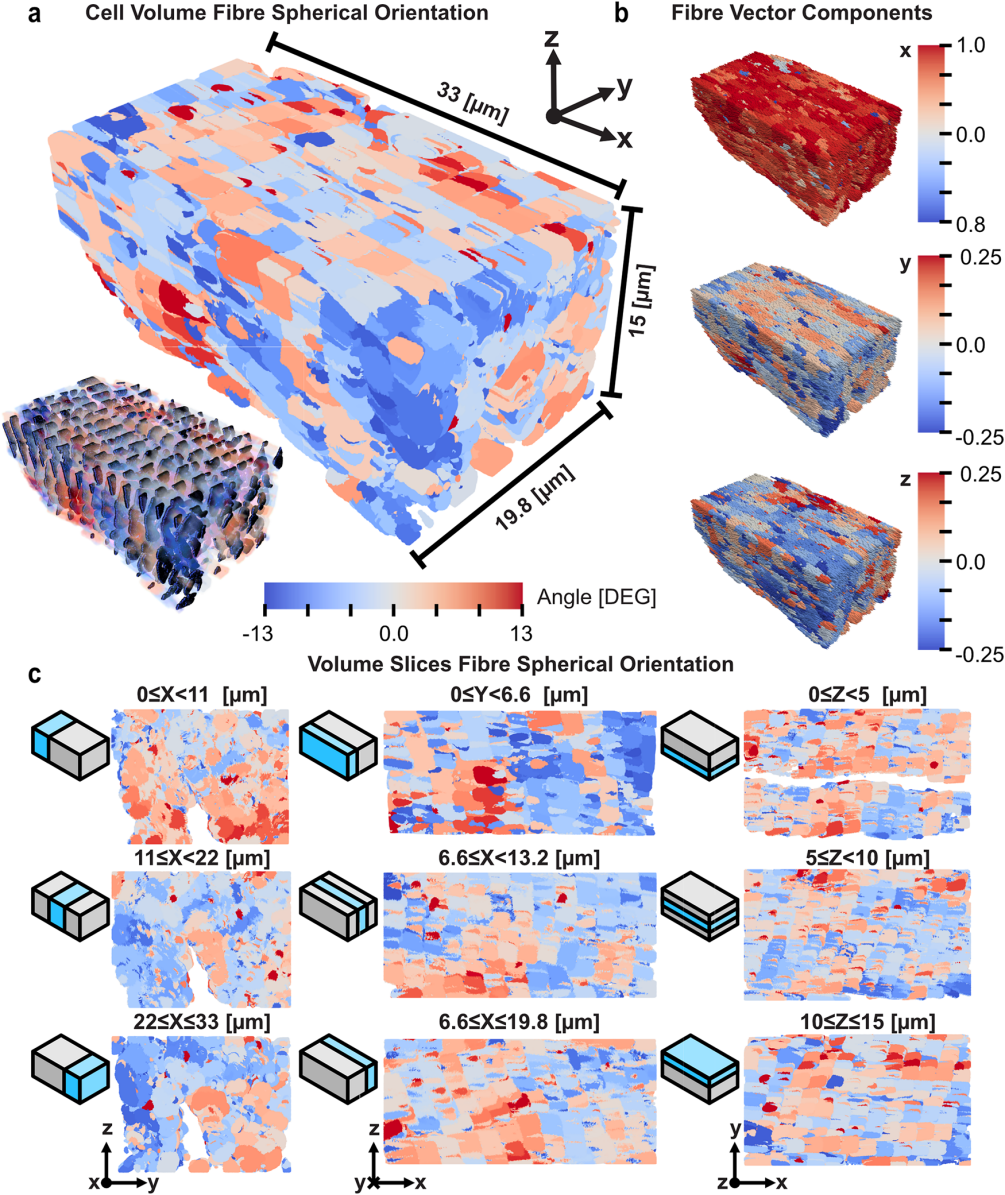
Interpolation of fibre orientation into cell volume. **a** Whole cell volume render with interpolation of z-disc orientations throughout lengths of sarcomere sections. The colour spectrum is limited to two standard deviations of global orientation values. Overlay of z-disc segmentations onto orientation render displayed in lower left. **b** Glyph render of cartesian vector components for each z-disc from top-to-bottom: $x$-component, $y$-component, $z$-component. $x$-component demonstrates homogeneity in principal direction as anticipated, $y$ and $z$ show larger variability. **c** Axial slices through the rendered volume. Each slice is a third of the dimension’s length. Note void in $x$-slices (left) which correspond with the large mitochondria group and nucleus observable in top-right $z$-slice. Slicing passes through z-discs and sarcomere unevenly, therefore z-disc patterning is less obvious.

The median of myofibril orientation also shifts across the cell volume, where Fig. 4a, c indicates that fibre orientation is heterogenous. Interestingly, slicing through the $x$-axis (Fig. 4c, left) depicts a transition from a positive deflection to a negative deflection. This observation mimics Fig. 3c, where Regions 0–8 and Regions 9–17 exhibit a decreasing trend in median orientation, the former being a larger gradient. The largest median positive orientations (R0, R9, R12—see Fig. 3c) all appear closer to the distal end ($0\le X < 11\,\mathrm{\mu m}$) of the cell, whereas the largest negative median orientations all occur on the proximal end ($22\le X\le 33\,\mathrm{\mu m}$; R6, R8, R17). Observing slices through the $y$-axis (Fig. 4c, middle) confirms this behaviour. Therefore, the cell proceeds from positive- to negative-median orientation shift over its length.

### Myofibril anisotropy introduces uneven deformation and rotation

A computational model of myofibril organisation and large nonlinear deformation mechanics was implemented using FEniCSx^48–51^ to investigate the influence of myofibril organisations on muscle contraction. Myofibril orientation was introduced into simulation meshes with an interpolation of orientation into mesh nodes. A Lagrangian framework was adopted following existing strategies for anisotropy^57^. Due to the Lagrangian formulation, a tensor push-forward transformation was used to convert the local coordinate space.

The change in coordinate space is mapped with the deformed contravariant, ${g}^{{ij}}$, and covariant, ${g}_{{ij}}$, metric tensors, where the convention of superscript (${\cdot }^{{ij}}$) and subscript (${\cdot }_{{ij}}$) is adopted for contravariant and covariant indices, respectively. From here, the Christoffel symbol of the second kind, ${\Gamma }_{{ij}}^{k}$, is calculated

1 $${\Gamma }_{{ij}}^{k}=\frac{1}{2}{g}^{{kl}}\left({g}_{{jl},i}+{g}_{{il},j}-{g}_{{ij},l}\right),$$

which characterises the change in coordinate space over a manifold. Here, a comma separated index (${\cdot }_{,i}$) indicates differentiation by that component. The Christoffel symbol then allows calculation of the covariant derivative, ${\nabla }_{j}\bullet$,

2 $${\nabla }_{j}{v}^{{\rm{\alpha }}}=\frac{\partial {v}^{\alpha }}{\partial {x}_{j}}+{\Gamma }_{{kj}}^{a}{v}^{k}.$$

The covariant derivative adjusts the regular derivative to the deformed space and forms a necessary part of variational form of the biophysical model with incompressibility restraint

3 $${\rm{R}}={\int }_{\Omega }{T}^{\alpha \beta }{F}_{{\rm{\beta }}}^{\,j}{\nabla }_{j}{v}^{{\rm{\alpha }}}d\Omega +{\int }_{\Omega }q\left(J-1\right)d,$$

where $T$ is the second Piola-Kirchhoff tensor, $F$ is the deformation gradient tensor, and $J$ is the third invariant, while $v$ and $q$ are the field variables. Contraction was simulated with stepwise displacement boundary conditions on the maximal and minimal faces of the mesh.

**Fig. 5:**
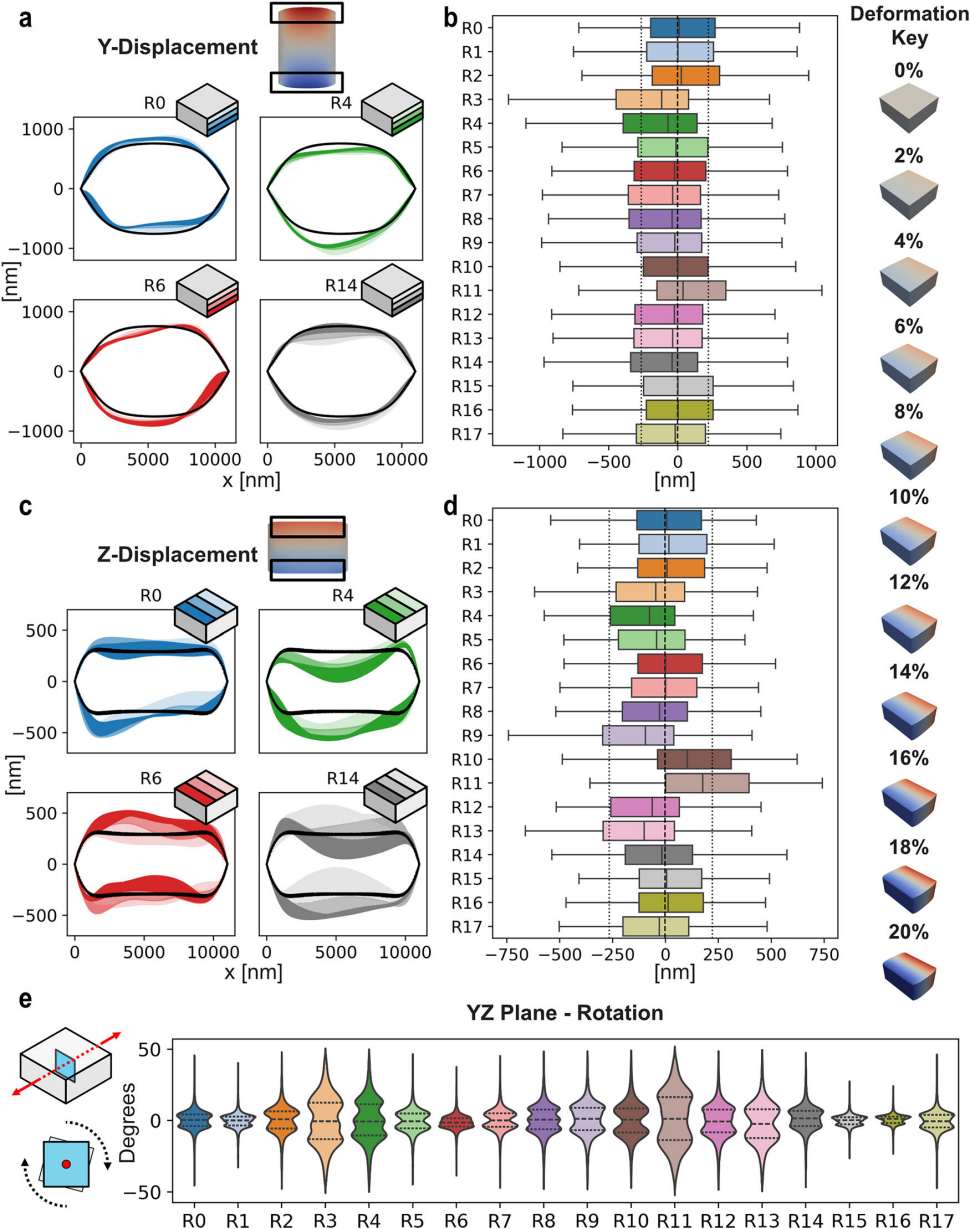
Region displacements and in-plane rotation at 20% displacement. Displacement visualised as select region shaded boundaries (**a**) and all regions boxplot distributions (**b**). **a** Boundary displacements for Regions $0$, $4$ 6, and 14 are overlayed onto displacements of uniaxial test. Displacements are plotted for thirds of the dimension to illustrate transition of deformation over length. Darkest shade indicates first slice, closest to origin ($y=0$). **b** Boxplots of each Region’s displacement values throughout volume. Dashed (--) and dotted (:) lines indicate quartiles of uniaxial test. **c**, **d** $z$-displacements visualised to similar capacity as (**a**, **b**). **e** ${yz}$-plane rotation produced by displacement displayed as violin plots with quartiles. Plane rotation determined at inner plane to reduce impacts of rigid boundaries. Violin plots show the distribution of common rotation values for each region, see Region 4 indicating frequent rotation values at $-{25}^{\circ },{\mathrm{0,25}}^{\circ }$.

Despite rigid displacement boundary conditions and incompressibility, myofibril anisotropy introduced asymmetrical and region-specific deformation. Comparison of the boundary $y$-displacement across mesh segments with the uniaxial deformation indicates that all test regions increase variance in deformation alongside a median shift (see Fig. 5a, b).

Four regions were randomly selected to more closely investigate how boundaries deform with anisotropy. Figure 5a, c plots the simulation mesh boundaries on $y$- and $z$-faces overlayed onto the boundaries of the uniaxial case (Fig. 5a, c; black solid lines). The simulation boundaries are displayed with three shaded sections depicting the nodal displacement in thirds of the volume due to their rapid changes over the surface. These regions depict how anisotropy deflects the volume’s maximum and minimum boundary irregularly in both the $y$- and $z$-axis. z-displacement in Regions 6 and 14 further indicate that despite a minimal median shift from the uniaxial case (Fig. 5d) there is notable deviation across the region’s profile; R14 begins with a predominantly negative deflection before progressing positively (see shading in Fig. 5c bottom right). Region 11 demonstrates the largest deflection in z-displacement, whilst also producing the lowest global alignment (see Supplementary Table 3) and subsequent large boundary deflections (see Supplementary Figs. 7 and 8). Therefore, despite volume constraints, introducing anisotropy creates boundary displacement deflections.

Myofibril anisotropy further introduces internal rotation. Measurement of rotation about the screw axis in the ${YZ}$-plane in our physiological sample exhibits unique profiles for all test cases (Fig. 5e). In contrast, the uniaxial case produces $0^\circ$ of rotation in the YZ-plane or across streamlines in the displacement field (see Supplementary Fig. 9). Internal rotation varies predominantly between $-25^\circ$ and $+25^\circ$, increasing with a larger median shift in displacement; direct comparison of scale of violin distribution (Fig. 5e) matches median shifting in displacement boxplots (Fig. 5d; for example, Regions 3, 4, 9, 10, and 11). Whereas more uniformly displaced tests that align medially with the uniaxial example demonstrate less rotation (i.e. Regions 6 and 16). Larger changes in deformation profiles due to anisotropy result in increased rotation of nodes within the mesh (see Supplementary Fig. 9).

### Anisotropy creates shear stress and unique spatial profiles

**Fig. 6:**
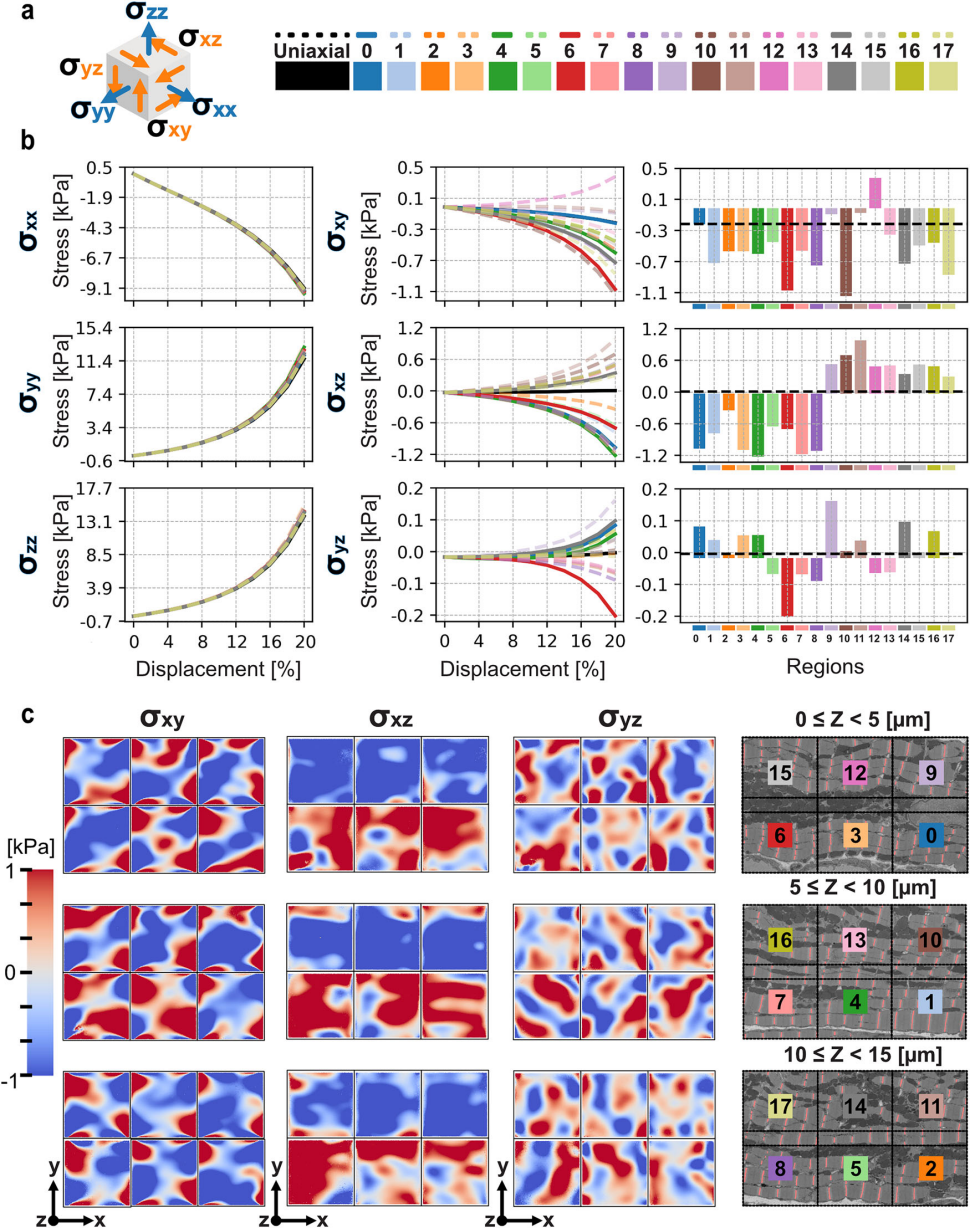
Stress trends and spatial shear for simulations at 20%. **a** (left) Diagrammatic visualisation of stress tensor components. (right) Test label key for stress trends and bar charts. **b** (left) Normal stress components plotted over displacement; nearly all test cases show minimal deflection from the uniaxial test, this is likely an artefact of rigid boundary displacement. Shear stress trend (middle) and bar charts (right) for each test condition. Shear trends demonstrate variance in shear profile as deformation reaches 20%. Bar charts are ordered numerically with region numbers, dashed (–) line indicates uniaxial test value. Values shown are final deformation stresses at 20%. **c** Spatial shear profiles for each region test case aligned with EM images of slices (z-discs in red). All distributions are displayed with the same colour bar range. Some pattern is evident in ${xz}$-shear which corresponds with barchart (**b**, middle).

Anisotropic myofibrils produce unique shear stresses not reproducible by a uniaxial model. The Cauchy stress tensor, ${\rm{\sigma }}$, (Fig. 6a; diagrammatic), calculated through conversion from the first Piola Kirchoff tensor, $P$, is decomposed and plotted for each displacement component in Fig. 6b.

4 $$P=J{\rm{\sigma }}{F}^{-T}+{pJ}{G}^{-1}{F}^{-T}$$

Where $F$ is the deformation gradient tensor, *p* is hydrostatic pressure, *G* is the undeformed metric tensor and indices with a subscript $T$, (${\cdot }^{T}$), indicating transpose.

The average normal stress components in each case are similar in response to the identical displacement boundary conditions for each simulation (Fig. 6b, left) and optimised to be within the bounds of experimental values^20,28,52–54^ (see Supplementary Fig. 11). However, all anisotropic simulations produce unique shear responses (Fig. 6b, middle and right). These profiles are most pronounced after $12 \%$ displacement, suggesting that anisotropy may be less relevant for early stages of contraction. Full contraction^3^, $20 \%$ (Fig. 6b right), demonstrates variability between all cases. Clearly, anisotropic regions, even with minimal orientation mean-shift, produce shear stress.

**Fig. 7:**
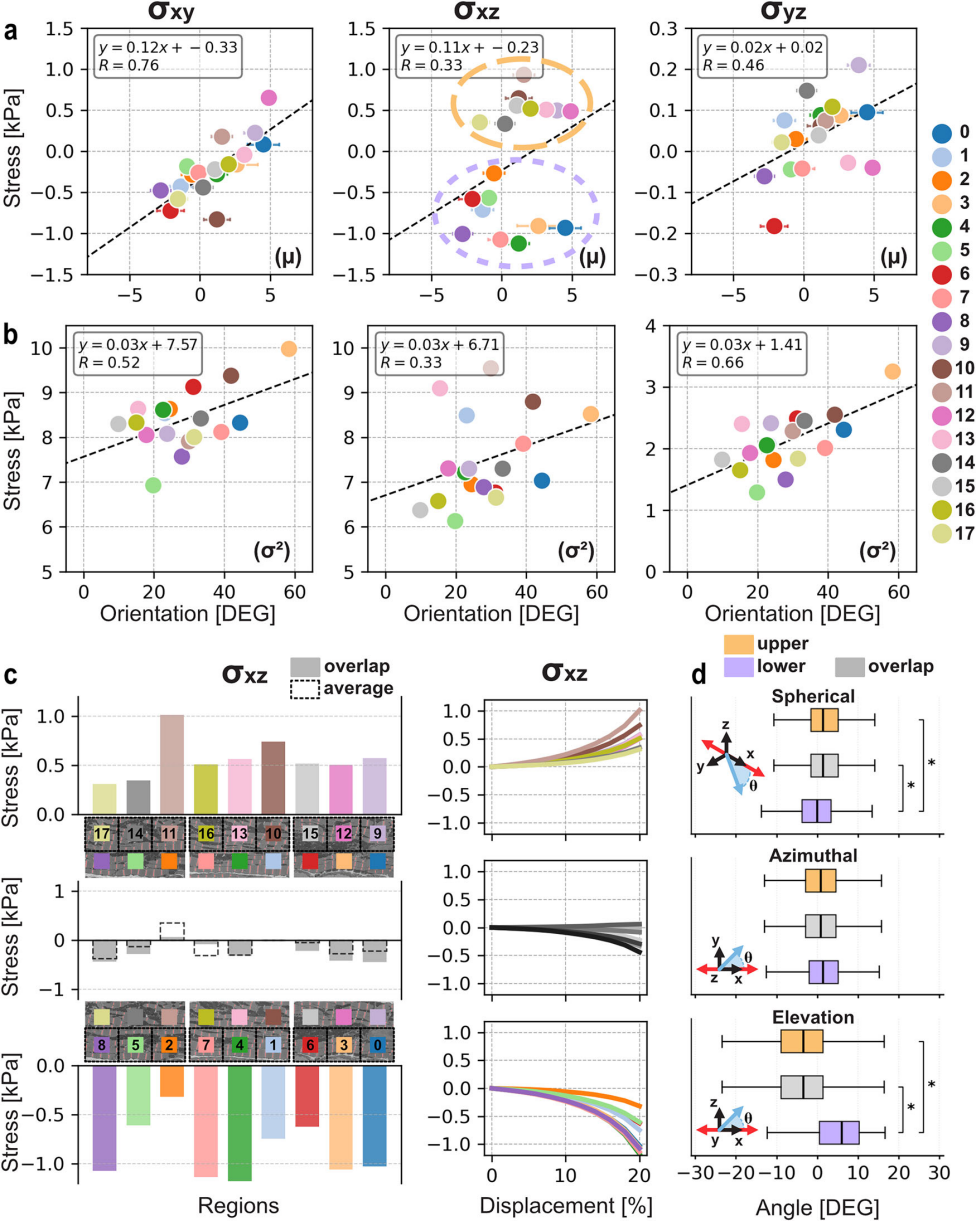
Trends for shear stress and z-disc orientation. **a** Mean shear stress versus mean z-disc orientation per region. (left) x-y shear stress produces strong positive correlation ($R=0.76$), increased positive mean orientation increases positive shear. (middle) x-z shear demonstrating a separation between shear in two groups with a weak positive correlation. Dashed circle (-) indicates upper regions ($y > 9.9\,\mathrm{nm}$), double dash (--) circle indicating group of lower regions ($y < 9.9\,\mathrm{nm}$), their correlations can be seen in Table 1. (right) y-z shear with moderate positive correlation. **b** Variance of shear stress versus variance of z-disc orientation. (left) x-y shear with moderate positive correlation. (middle) x-z shear with weak positive correlation. (right) y-z shear with moderate positive correlation. **c** Bar charts and trends of shear stress (x-z) for each region. (top) Upper region ($y > 9.9\,\mathrm{nm}$) shear shows positive behaviour in both final value bar charts and trends. (middle) Overlap region simulations (solid) demonstrates that shear behaviour transitions over space. Average shear between upper and lower simulations (dashed, [–]) overlayed on bar chart. (bottom) Lower region ($y < 9.9\,\mathrm{nm}$) shear stress is negative for all simulations and during trend. **d** Comparison of angle orientations across Upper, Lower, and Overlap regions, pairwise t-test significance is indicated with asterisk (*, $p < 0.05$). (top) Spherical deviation, as previously reported, indicates lower regions are more negatively oriented than other regions. (middle) Distributions of azimuthal (lateral, x-y) angle indicates no significant differences between regions. (bottom) Elevation (x-z) angle demonstrates that lower regions have significantly more positive orientation.

Shear stress in the ${xz}$- and ${yz}$-components is distributed between positive and negative inflection for simulations (Fig. 6b, right). In contrast, ${xy}$-shear was decidedly negative for all but one test, R12, which had a large positive skew in orientation distribution (see Fig. 3c); ${xy}$-shear was also the only non-zero shear for the uniaxial case. This non-zero shear for the uniaxial simulation is likely due to the artificial degree of freedom constraint on the $x$-axis faces. Normalising for the uniaxial case results in four simulations with positive stress (see Fig. 7a), though this may still be artificially constrained. Implementation of deformation with active contraction, however, shows that the produced shear profiles are comparable with the artificially constrained boundaries (see Supplementary Fig. 11). ${xz}$- and ${yz}$-shear, alternatively, produce null shear in the uniaxial case with notable distribution ratios of positive and negative shear stress for all simulations (${xz}$: $1:1$, ${yz}$: 8:6, ${positive}:{negative}$ for tests with noticeable shear).

Shear stress exhibits a spatial relationship over the cell volume like that observed in the orientation behaviour. The balance of shear stress displayed by the ${xz}$-component, visualised by the bar charts in Fig. 6b, corresponds with the spatial shear distributions (Fig. 6c). ${\sigma }_{{xz}}$ is noticeably negative for all upper regions of the cell slices (Fig. 6c left), with a positive value for all lower slices. This effect is most apparent for Regions 0–2 and 9–11 which oppose sides of a nucleus.

Analysing the relationship between orientation and shear stresses demonstrates a positive correlation between spherical deviation and shear (Fig. 7). All shear tests present a positive correlation, however, for whole-group analysis the strongest positive relationship is observed for ${xy}$-shear ($R=0.76$, Fig. 7a left). ${xz}$-shear, however, produces two separate clusters of points (Fig. 7a middle) which match the positive and negative shear group described above for upper regions ($y > 9.9\,\mathrm{nm}$) and lower regions ($y < 9.9\,\mathrm{nm}$). Regression analysis of spatial clusters is present in Table 1. The largest correlation between ${xz}$-shear and mean orientation was found for the highest spatial cluster ($z > 10\,\mathrm{nm}$). This cluster exhibits myofibrils which are uninterrupted by large mitochondrial groups or nuclei, unlike lower clusters (see Figs. 2b–d and 4c). Similarly, the group produces a stepwise increase in both mean orientation angle (see Fig. 3c) and average shear stress (see Fig. 7c) along its length, with R8 and R11 displaying the largest pairwise difference in ${xz}$-magnitude. Variance is also positively correlated between stress and orientation.

**Table 1:** Comparisons of grouped trends and correlations for shear stress (${\sigma }_{{xy}},{\sigma }_{{xz}},{\sigma }_{{yz}}$) versus orientation ($\theta$)

Group		${\sigma }_{{xy}}$		${\sigma }_{{xz}}$		${\sigma }_{{yz}}$	
		${\bar{\sigma }}_{{xy}}(\bar{\theta })$	$R$	${\bar{\sigma }}_{{xz}}(\bar{\theta })$	$R$	${\bar{\sigma }}_{{yz}}(\bar{\theta })$	$R$
All regions		${\bf{0}}{\boldsymbol{.}}{\bf{12}}\bar{{\boldsymbol{\theta }}}-{\bf{0}}{\boldsymbol{.}}{\bf{86}}$	${\bf{0.76}}$	${\bf{0.11}}\bar{{\boldsymbol{\theta }}}-{\bf{0.30}}$	${\bf{0.33}}$	${\bf{0.02}}\bar{{\boldsymbol{\theta }}}-{\bf{0.03}}$	${\bf{0.46}}$
Across length							
	$x\ge {\boldsymbol{22}}\,{\bf{nm}}$	${\bf{0}}{\boldsymbol{.}}{\bf{10}}\bar{\theta }-{\bf{0}}{\boldsymbol{.}}{\bf{87}}$	${\bf{0}}{\boldsymbol{.}}{\bf{86}}$	${\bf{0}}{\boldsymbol{.}}{\bf{25}}\bar{{\boldsymbol{\theta }}}-{\bf{0}}{\boldsymbol{.}}{\bf{13}}$	${\bf{0}}{\boldsymbol{.}}{\bf{62}}$	${\bf{0}}{\boldsymbol{.}}{\bf{04}}\bar{{\boldsymbol{\theta }}}-{\bf{0}}{\boldsymbol{.}}{\bf{04}}$	${\bf{0}}{\boldsymbol{.}}{\bf{76}}$
	${\bf{11}}\ge x\, < \,{\bf{22}}\,{\bf{nm}}$	${\bf{0.14}}\bar{{\boldsymbol{\theta }}}-{\bf{0.87}}$	${\bf{0.80}}$	${\bf{0.14}}\bar{{\boldsymbol{\theta }}}-{\bf{0.55}}$	${\bf{0.41}}$	$-{\bf{0.01}}\bar{{\boldsymbol{\theta }}}+{\bf{0.01}}$	$-{\bf{0.31}}$
	$x\, < \,{\bf{11}}\,{\bf{nm}}$	${\bf{0.11}}\bar{{\boldsymbol{\theta }}}-{\bf{0.87}}$	${\bf{0.60}}$	${\bf{0.03}}\bar{{\boldsymbol{\theta }}}-{\bf{0.09}}$	${\bf{0.09}}$	${\bf{0.02}}\bar{{\boldsymbol{\theta }}}-{\bf{0.02}}$	${\bf{0.66}}$
Across width							
	${\boldsymbol{y}}\, > \,{\bf{9.9}}\,{\bf{nm}}$	${\bf{0.19}}\bar{{\boldsymbol{\theta }}}-{\bf{1.00}}$	${\bf{0.80}}$	${\bf{0.02}}\bar{{\boldsymbol{\theta }}}-{\bf{0.44}}$	${\bf{0.17}}$	${\bf{0.00}}\bar{{\boldsymbol{\theta }}}+{\bf{0.03}}$	$-{\bf{0.08}}$
	${\boldsymbol{y}}\, < \,{\bf{9.9}}\,{\bf{nm}}$	${\bf{0.08}}\bar{{\boldsymbol{\theta }}}-{\bf{0.83}}$	${\bf{0.84}}$	$-{\bf{0.04}}\bar{{\boldsymbol{\theta }}}-{\bf{0.86}}$	$-{\bf{0.33}}$	${\bf{0.03}}\bar{{\boldsymbol{\theta }}}-{\bf{0.04}}$	${\bf{0.70}}$
Across depth							
	${\boldsymbol{z}}\ge {\bf{10}}\,{\bf{nm}}$	${\bf{0.14}}\bar{{\boldsymbol{\theta }}}-{\bf{0.73}}$	${\bf{0.76}}$	${\bf{0.39}}\bar{{\boldsymbol{\theta }}}+{\bf{0.64}}$	${\bf{0.81}}$	${\bf{0.04}}\bar{{\boldsymbol{\theta }}}+{\bf{0.01}}$	${\bf{0.74}}$
	${\bf{5}}\ge {\boldsymbol{z}} < {\bf{10}}\,{\bf{nm}}$	${\bf{0.07}}\bar{{\boldsymbol{\theta }}}-{\bf{0.93}}$	${\bf{0.40}}$	${\bf{0.35}}\bar{{\boldsymbol{\theta }}}+{\bf{0.15}}$	${\bf{0.66}}$	${\bf{0.00}}\bar{{\boldsymbol{\theta }}}-{\bf{0.00}}$	$-{\bf{0.11}}$
	${\boldsymbol{z}}\, < \,{\bf{5}}\,{\bf{nm}}$	${\bf{0.16}}\bar{{\boldsymbol{\theta }}}-{\bf{0.96}}$	${\bf{0.92}}$	${\bf{0.05}}\bar{{\boldsymbol{\theta }}}-{\bf{0.34}}$	${\bf{0.18}}$	${\bf{0.03}}\bar{{\boldsymbol{\theta }}}-{\bf{0.09}}$	${\bf{0.67}}$

*Note:* ${\boldsymbol{R}}$ is Pearson’s correlation coefficient, $\bar{{\boldsymbol{\sigma }}}$ represents mean stress, $\bar{{\boldsymbol{\theta }}}$ represents mean orientation.

To further investigate the spatial relationship of ${xz}$-shear, simulations were run on the overlap between upper and lower regions (Fig. 7c). Clustering shear by upper, overlap, and lower groups, clearly indicates the spatial relationship of ${xz}$-shear direction. All upper regions producing positive shear, and all lower regions producing negative shear. If the simulated shears could be physically coupled, then it was hypothesised that overlapping regions should produce shear profiles that demonstrate a transition between these extremes. Accordingly, ${xz}$-shear for all overlap regions was significantly lower in magnitude. The direction of shear is also directly related to whichever (upper or lower) adjacent region has a larger magnitude. Similarly, overlaying the average shear between the two clusters onto the overlap simulations (Fig. 7c middle) indicates further that despite being simulated in isolation the shear behaviour can be related between adjacent regions.

Investigating the orientation behaviour of the upper, overlap, and lower regions indicates significant difference in profiles. Figure 7d shows the orientation distributions of z-discs within each cluster for spherical, azimuthal, and elevation angles. Whilst azimuthal angles show no significant difference between the groups, spherical deviation of the lower cluster is different (t-test, $p < 0.05$) from both the overlap region and the upper region. Similarly, elevation angle shows the same differentiation with a much larger change in median and distribution (Fig. 7d bottom). Elevation angle (x-z) would also directly influence ${xz}$-shear, with the significant difference in median angle corresponding with the opposing shear profiles.

## Discussion

This study reveals that cardiac muscle cell myofibrils create a distribution of orientations and produce unique intracellular shear stress distributions. This finding challenges long-standing assumptions based on a uniaxial model, which has treated muscle contraction as occurring purely under axial loading since Huxley’s foundational work in the early 1900s^16^, as well as more contemporary ultrastructural imaging work^1^, biophysical simulations of fibre force^23,27^, and experimental measurements^19,58^.

Understanding the production of shear stresses has the potential to elucidate the impacts of multi-axial force trajectories; existing biophysical models have so far limited active stress dynamics to uniaxial forces. However, lateral force transmission is an important feature of musculoskeletal physics^25,59^ and has burgeoning appreciation in cardiac constitutive models^60,61^. Recent work has demonstrated that lateral forces change in disease states^62^ and theorise its role in inter-cellular transmission^59,63^. Further, Rothermel et al. ^64^ demonstrated that transverse length-tension relationships in neonatal cardiomyocytes follow the same trend as axial tension, which they hypothesise is a result of myofilament spacing. Similarly, the shear stress trends calculated here (Fig. 6) follow the same exponential behaviour as axial measurements^19,20,53,65^, which may represent a combination of the radial myofilament spacing Rothermel et al. ^64^ proposed and cardiomyocyte passive viscoelastic properties^66^. While historical models of myofibril arrangement do not characterise these dynamics, our physiologically informed biophysical model demonstrates the presence and magnitude of shear stresses that would contribute to multi-axial force.

Local shear may influence mechanosensitive processes. Recent work by Rog-Zielinska et al. ^43,67^ has demonstrated that both the mitochondria and t-tubules deform during contraction with the latter experiencing an increase in metabolite diffusion during contraction. Both structures are local to myofibrils^55^ and the spatial shear (Fig. 6) and deformation patterns (Fig. 5) simulated here would likely influence their behaviour. Similarly, shear behaviour may contribute to the motility of nuclei during development^42^, which was suggested by previously reported helical myofibril structures^33^. Bavi et al. ^68^ simulated the influence of local membrane curvature on mechanosensitive channels and found that these structures are pro-activation. Caveolae on mitochondrial membranes^40^ may therefore be an interesting recipient of local-level shear. Likewise, titin is known to respond to stress and modulate myocyte hypertrophy^69^. Converting the simulated axial and shear stresses to force per titin molecule, similar to Granzier and Irving^20^, demonstrates these values fall within the expected range for sarcomere strain (ranges $2$–$4\,\mathrm{pN}$ and $0.05$–$0.4\,\mathrm{pN}$)^20,70^. How shear impacts hypertrophic signalling remains unclear and warrants investigation. Piezo1 similarly modulates hypertrophy and is known to be mechanosensitive^71^. Previous reports have demonstrated that activation can occur on locally deforming membranes at a pico-Newton ($\mathrm{pN}$) scale^68,72^, though the shear forces here are likely too low to activate Piezo1^73^, further testing may help elucidate the coupling between mechanosensitive channels and shear.

Visualisation of the spatial shear stress relationship over isolated cell regions, and observations of internal rotation, indicates that myofibrils produce local torsions under deformation. As displayed in Fig. 5e, all simulations experienced internal rotation due to anisotropy. Similarly, in Fig. 6c shear in the ${xz}$-component indicates that lateral sides of the cell experience opposing shear. This behaviour was then explored by simulating the overlapping regions between the lateral sides and observing that shear stress transitions from peak negative and positive magnitudes across the cell centre, with overlap regions producing shear approximating the average of either adjacent region (Fig. 7c). Whilst the regions are still physically insulated, this transition suggests that local shear simulations can provide inference on adjacent cell sections. Further, comparison with active contraction (see Supplementary Fig. 11) demonstrates similar shear behaviour, though revealing that passive deformation may underestimate the magnitude. However, a larger-scale analysis is still required to understand whole-cell shear behaviour within its tissue context.

Shear stress demonstrates a correlation with orientation angle (Fig. 7a). As z-disc spherical deviation angle increased, all shear responses similarly increased, with the strongest correlation being ${xy}$-shear (Table 1). When clustering the lateral sides and comparing their z-disc elevation orientation, the two sides were shown to differ significantly in spherical and elevation angles. Whilst the cell has no discrete axis for defining elevation or azimuth, measurements relative to the cell’s fixation during imaging highlight that the lower and upper regions differ significantly in elevation which may explain their opposing shear profiles. This bilateral shear and orientation behaviour suggests that anisotropy would produce internal rotation in isolated regions during contraction, and more broadly that shear stress may produce torsion about the cell’s major axis. Cardiomyocyte torsion would interestingly mimic ventricular twisting during systole^74^ suggesting further multiscale mechanical coupling and warranting further investigation.

Reports have demonstrated that muscle cell architecture is impacted by fibre type^15,34^. Cardiomyocytes require a constant provision of energy dissimilar from skeletal muscle, impacting the size, orientation, and connectivity of mitochondria and sarcomeres^34^. Similarly, Willingham et al. ^15^ displayed that branching events in myofibrils are increased in slow-twitch muscles compared to cardiac and fast-twitch variants, whereas Ajayi et al. ^35^ indicated that myofibril branching is impacted by gene knock-out in *Drosophila*. This work demonstrates that non-axial orientation produces shear stresses significantly different from the uniaxial case in a physiological model of a cardiomyocyte regions, future work, therefore, may focus on the whole cell, or how other muscle cell architectures impact shear stress. Further, recent studies have indicated that multiscale models can inform how physics at a cellular level influences bulk tissue^75,76^, pivotal to observing remodelling in disease. Myocyte architecture is known to change in pathology^77,78^, therefore modelling the impacts of these changes on shear and its compounding effects on tissue should be investigated.

Characterisation of myofibrils in other cells indicate the applicability of these findings beyond the single cardiomyocyte. Quantification of networks has indicated that branching^15,35^ and orientation^56^ distributions are standard in cardiomyocytes. The orientation data calculated here (see Fig. 3) is similar to distributions previously reported^56^. Similarly, analysis of another cell section indicated that orientation distribution, mean, and standard deviation, all fall within the current distribution (see Supplementary Fig. 5). Therefore, it is anticipated that the local shear behaviour explored here is representative of local region in other cardiomyocytes. Jayasinghe et al. ^33^ showed that myofibrils create helices that form over the cell nucleus, a feature that would complement the centripetal forces required for nuclei migration described by Roman et al. ^42^, for this reason it is expected that these regions experience shear and torsion behaviours similar to described here. Further, the cytoskeleton has been shown to modulate shear stiffness in cardiomyocytes^79,80^. Nishimura et al. ^79^ demonstrated that microtubule stiffness, increasing with disease, produces shear stresses during experimentation and in FE studies with uniaxial myofibrils. It is likely that cytoskeleton and multi-axial stress present here couple during contraction.

The hyperplastic anisotropic biophysical model presented here is a foundational model which has the further potential to incorporate other cell physiology. First, myofibril orientation is established perpendicular to z-discs and then smoothed between subsequent z-discs (see Methods) to approximate myofilaments splines. Whilst myofilaments are anchored at the z-disc structurally^81^ and have been used as a proxy for myofibril architecture in cardiomyocytes previously^33^, it is the myofilaments which dictate force. Myofilaments have been shown to curve over the sarcomere, though this is minimal in cardiac muscle^55^. Further, this model does not consider splitting events which, if originating at the z-disc as suggested^32^, would likely increase local shear as myofilaments diverge from the z-disc. Myofilaments also form complex binding structures^55,82^, producing helictical and triangular lattices over the length which may influence force dynamics. Active contraction behaviour, and calcium dynamics, are also important contributions that have been incorporated into other multi-dimensional cell models^26,27,83^; incorporation of these components could be used to produce a more detailed physiological model. Further, changes to the rigid boundary conditions considered here may impact how extreme anisotropy deviates from the uniaxial model, with emphasis on coupling physics to expand insight across simulated regions and local organelles.

In this study, we investigated, to our knowledge, the largest and most detailed z-disc segmentation of a cardiomyocyte and produced the first fibre-orientation informed non-linear myocyte deformation model. Deep learning segmentation of the cardiac cell confirmed that z-disc produces a distribution of orientation values concentrated about the major axis. These values varied up to $30^\circ$ and were not significantly impacted by organelles. However, fibre orientation did display characteristic differences across the cell geometry with a gradient of positive to negative mean-shift over the cell length.

Myofibril anisotropy produces shear stresses that are not reproducible with the uniaxial model. Whilst normal stresses were maintained, physiologically informed simulations of cell regions created arrays of shear values. All simulations produced shear components of greater magnitude than the uniaxial case, the latter resulting in no shear in the ${xz}$- and ${yz}$-components. This study indicates that incorporating off-axis myofibril orientation is necessary to account for local shear stresses produced during cardiac muscle cell contraction.

## Methods

### Z-disc segmentation

The ultrastructure of sheep left ventricular cardiomyocytes was captured with scanning-block-face electron microscopy (SBF-EM) as previously described^75^. Within this volume ($6000\times 6000\times 359\,\mathrm{pixels};\,X\,{and}\,Y:\,0.11\frac{\mathrm{\mu m}}{\mathrm{pixe}l},\,Z:0.5\frac{\mathrm{\mu m}}{\mathrm{pixel}}$), three cardiomyocytes were present and 19 random regions ($1024\times 1024\times 100[{pixels}]$) were isolated for manual annotation of z-discs in Napari^84^. These annotations were then used to automatically segment the largest cardiomyocyte. The chosen cardiomyocyte was a full cell depth and half-length of similar cardiomyocytes ($\sim 94\,\mathrm{\mu m}$)^85^.

A smaller initial subset of these patches was annotated and used to train a 2D U-Net++^47^ with a ResNet-50^86^ weights using unified focal loss^87^. The output segmentations from this original model were used to create a segmentation on the remaining patches which were manually corrected before creating the full set to train the final model. During each epoch smaller patches from the $19$ annotated 3D blocks were extracted to introduce training set variability. Sampled 2D patches were $384\times 384$ pixel covering $70 \%$ area, resulting in $\mathrm{1,648}$ patches per epoch.

Output segmentation voxels were generated through 2D smoothing tiling of overlapping predictions. Manual corrections were made to erroneous segmentations after inspection and before utilisation in further analysis. In total $1138$ z-discs were segmented and included in this study, with manual annotation regions comprising ~$14.9 \%$ of the whole volume and automatic segmentation comprising $\sim 59.9 \%$.

### Morphological analysis

For morphological analysis, orientations were normalised to the cell’s contraction axis. The contraction axis was determined as the first principal component of the cardiomyocyte, its long axis. The contraction axis was validated via the average orientation angle of the z-discs (see Fig. 3); the average orientation aligned with the contraction axis. This is reminiscent of similar orientation measurements^56^. From here, instances of z-discs were analysed to inform on centroid position, pixel-size, and orientation. Centroid data, pixel quantities, size, and elliptical axis were calculated with the scikit-image^88^ *measure* module.

Fibre orientation was defined through the z-discs’ normal vector in direction of cell’s major axis. To determine the normal vector principal components analysis (PCA)^89^ was utilised; the first two components characterising the structure of the segmentation, as the major and minor axis of length and width. The third component is, by definition, perpendicular to the first two and normal to the face. PCA was implemented with scikit-learn^90^.

The spherical orientation was calculated as the angle between the vectors of the third principal component and contraction axis (see Fig. 3a). To achieve this all third components were required to be pointing in the positive *x*-axis. The resulting distribution of values is displayed in Fig. 3b with a bin size of $1.5^\circ$. Bin sizes larger than this are insufficient for appreciating variance, smaller sizes increase noise.

### Mesh

A prism mesh defined on the dimensions of cell regions was created in Gmsh^91^. The geometry ($11,000\times 11,000\times 5000\,\mathrm{nm}$) was defined with second-order tetrahedral elements. Mesh refinement was tested to ensure convergence of solution and minimisation of adjacent node difference. The resulting test mesh had $\mathrm{358,656}$ elements and $\mathrm{463,120}$ nodes.

### Constitutive equation

The Guccione type constitutive equation (see Eq. 1) for orthotropic behaviour was implemented^61^.

5 $$\begin{array}{l}\Psi =\frac{{c}_{1}}{2}\,* \,{(e}^{{\rm{Q}}}-1)\\ Q={b}_{f}{E}_{{ff}}^{2}+{b}_{t}\left({E}_{{tt}}^{2}+{E}_{{ss}}^{2}+{E}_{{ts}}^{2}+{E}_{{st}}^{2}\right)+{b}_{s}\left({E}_{{fs}}^{2}+{E}_{{sf}}^{2}+{E}_{{tf}}^{2}+{E}_{{ft}}^{2}\right)\end{array}$$

Where $\Psi$ is the strain energy density function, and $E$ is the green Lagrange strain tensor with components $f,t,s$ are the tensor components.

Here, ${b}_{t}$ and ${b}_{s}$ were set as equal reducing the equation to transverse isotropic as previously explored^21,92^. The other constants (${b}_{f}$, ${b}_{t}$, ${c}_{1}$) were calculated via optimisation against experimental and model trends of tension development in cardiomyocytes. The literature presents a range of values so $9\,\frac{\mathrm{kN}}{{\mathrm{mm}}^{2}}$ (or ${kPa}$) was set to balance the range of experimental measurements of cardiac tension^20,28,52–54^. Solutions converged to ${b}_{f}\approx 12.68\,{kPa}$, ${b}_{t}\approx 11.04\,{kPa}$, ${c}_{1}\approx 2.81.$

### Regions orientation interpolation

Myofibril orientation was interpolated into the mesh by first mapping the nodal positions of the mesh to the z-disc pixel data from segmentation regions. This was supported with SciPy’s^93^ KD-tree. Once mapped, angle data was interpolated into second-order ($10$-node) tetrahedral Lagrange functions with the in-built FEniCSx^48–51^ architecture. Nodes within the z-disc boundaries were assigned to the same angles as those which were directly mapped to the nearest-neighbour tree.

Force production in muscle cells occurs in the direction of the myofilaments. This model assumes that myofilament orientation can be tracked between subsequent z-discs. Imaging has demonstrated that cardiac muscle myofilaments diverge minimally from a linear path compared to other fibres^55^. To ensure continuity over the sarcomere, Gaussian smoothing of orientation was applied between z-discs. Smoothing boundaries were based on the diameters of z-disc (disc-axis) and length of sarcomeres (fibre-axis). Reports suggest cardiac z-disc range from $100$ to $140\,\mathrm{nm}$^94,95^, and sarcomere slack length of $1.8-2\,\mathrm{\mu m}$^1,3,96^; these values corresponded to the standard deviations provided to the smoothing function.

### Fibre field

Anisotropy was incorporated into the variational calculus similarly to Nash and Hunter^57^ and Guccione et al. ^61^, following the Lagrangian formulation. First, undeformed basis vectors (${A}_{i}$) and metric tensors (${G}_{{ij}}$) are calculated via rotation of cartesian coordinates to orient with the myofibril data in each region. Here, ${G}_{{ij}}$ is the inner product of the basis vectors,

6 $${G}_{{ij}}={A}_{i}\cdot {A}_{j}$$

Where $A$ represents the Lagrange basis vectors. This is interpolated into Quadrature points within native FEniCSx^48–51^. FEniCSx provides the Lagrangian form of variational calculus, therefore the Euclidean metric covariant and contravariant tensors are also determined. Lower case ${g}_{{ij}}$ and ${a}_{i}$ are used as convention to indicate deformed tensors.

7 $${g}_{{ij}}={a}_{i}\cdot {a}_{j}$$

Subsequentially, the Christoffel Symbols of the second kind,

8 $${\Gamma }_{{ij}}^{k}=\frac{1}{2}{g}^{{kl}}\left({g}_{{jl},i}+{g}_{{il},j}-{g}_{{ij},l}\right),$$

and covariant derivatives,

9 $${\nabla }_{j}{v}^{{\rm{\alpha }}}={v}_{,j}^{{\rm{\alpha }}}+{\Gamma }_{{kj}}^{a}{v}^{k},$$

are calculated to update the variational form

10 $${\rm{R}}={\int }_{\Omega }{T}^{\alpha \beta }{F}_{{\rm{\beta }}}^{j}{\nabla }_{j}{v}^{{\rm{\alpha }}}d\Omega +{\int }_{\Omega }q\left(J-1\right)d.$$

The First-Piola Kirchoff is calculated per a push-forward transform on the Cauchy stress tensor.

11 $$P=J{\rm{\sigma }}{F}^{-T}+{pJ}{G}^{-1}{F}^{-T}$$

### Boundary conditions and simulation

Contraction was simulated with discrete displacement of the regions at maximum and minimum $x$-position. The planes were fixed in $y$- and $z$-axes with the $x$-axis displaced $20 \%$. All other degrees of freedom move freely.

An iterative non-linear solver native to FEniCSx^48–51^ was employed. The solution was incremented at 2% displacement intervals. Convergence was determined with an incremental tolerance calculation, requiring a tolerance of ${10}^{-5}$ and a maximum of $50$ iterations per Newton Solver. Simulations were run on the University’s High Performance Computer and were allocated 512GB per node and run serially to avoid node-mismatch in parallel.

### Displacement and rotation

Boundary displacements for each region were compared by taking the edges of the deformed shape. To compare $z$-displacement, values along $x$-axis were plotted for three regions through the $y$-axis. Maximum and minimum displaced values on the upper and lower edge of these regions were overlayed (see Fig. 5). Similarly, $y$-displacement was displayed across the $x$-axis for three regions across the $z$-axis.

Rotation data were extracted from the centre region and calculated in the $y$-$z$ plane about $x$-axis. Two vectors were produced once each datapoint was centralised to the origin: the first being a vector to the undeformed point, the second being to the deformed point. The angle difference between the two with the $y$-axis was provided as the angle of rotation.

### Stress and strain calculation

Stress values were extracted from an internal $90 \%$ volume. This bounding allowed for the reduction of boundary artefact in the solutions and overestimations of stress. Cauchy stress was calculated with the inversion of Eq. 7 yet omitting hydrostatic pressure. Hydrostatic pressure was interpolated with first-order (4-node) Lagrange tetrahedra.

Each tensor component was then calculated by taking the mean stress over the internal volume in each direction. The stresses displayed in Fig. 6 correspond with the principal directions and relevant shear terms. All spatial visualisations were achieved in Paraview^97^.

## Supplementary information

Supplementary information

## Data Availability

Raw microscopy data used in this study are from a prior publication and are available upon request. Segmentation outputs and codes for simulation and analysis are available on our publicly accessible GitHub repository at https://github.com/CellSMB/CardiacMyofibrilsInduceShearStress.git.
