# Supplementary material for: Cardiac myofibril networks induce shear stress
Source: NPJ Syst Biol Appl. 2026 Apr 2;12:75. doi: 10.1038/s41540-026-00696-1 (PMC13216349; doi:10.1038/s41540-026-00696-1)
Supplement: Supplementary file 1 — Supplementary information [file 41540_2026_696_MOESM1_ESM.pdf]

Supplementary Figure 1

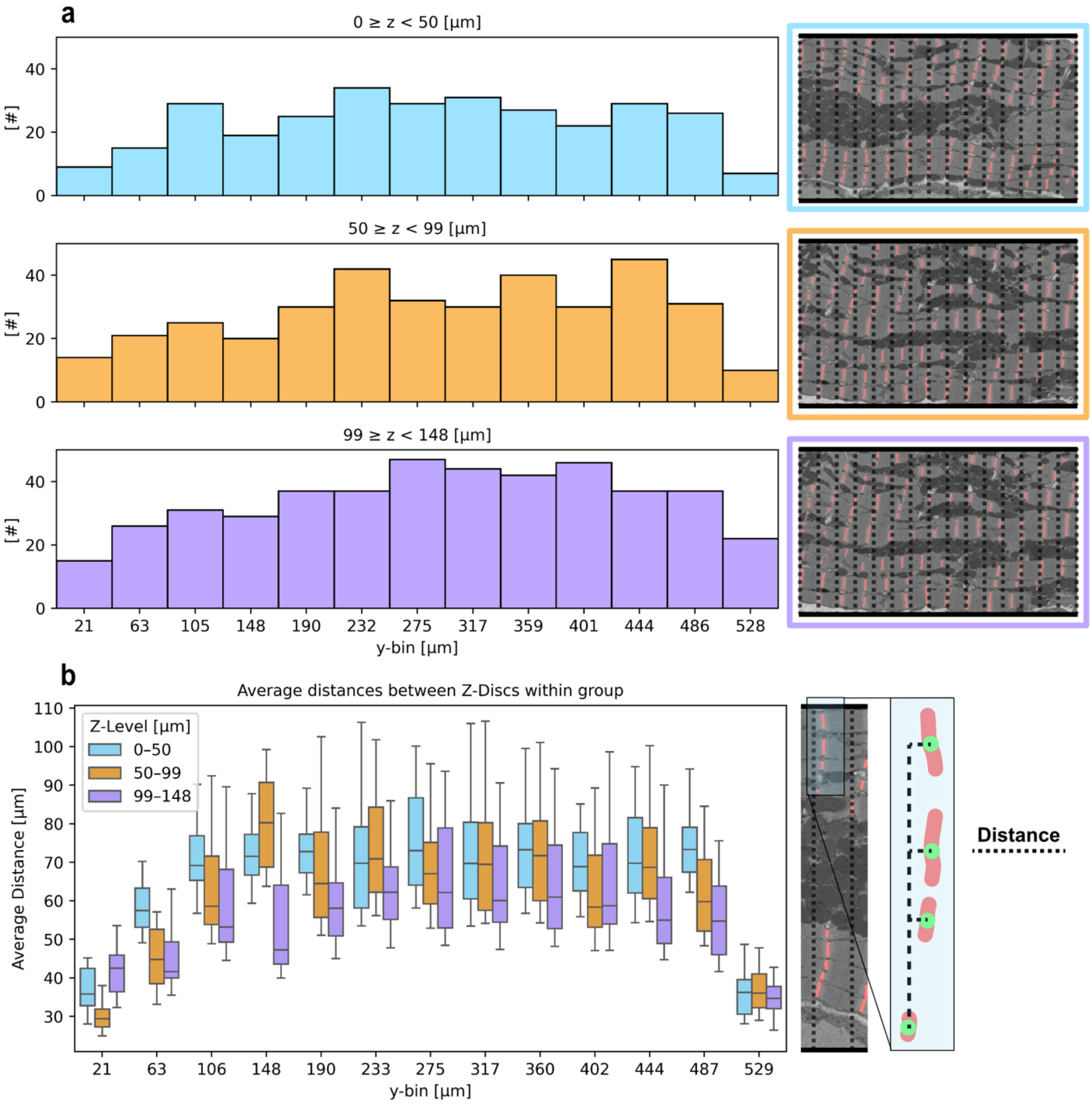

Distances between grouped Z-Discs across cell width. (a, left) Histograms of Z-Discs centroids across three difference z-axis slices. (right) EM images of Z-Disc (in red) regions indicative of corresponding histogram; overlay of dashed (--) lines to represent boarder of bins in histograms. Distributions show similarly of shape as Z-Discs across cell fall within bins. Each slice demonstrates a similar quantity of Z-Discs. (b, left) Boxplots of inter-bin distances between each centroid (see right for indication) and those within the same group. Smaller groups have tighter distribution of distances due to decreased sample sizes. Boxplot spread indicates Z-Discs centroids are not aligned over cell width.

Supplementary Figure 2

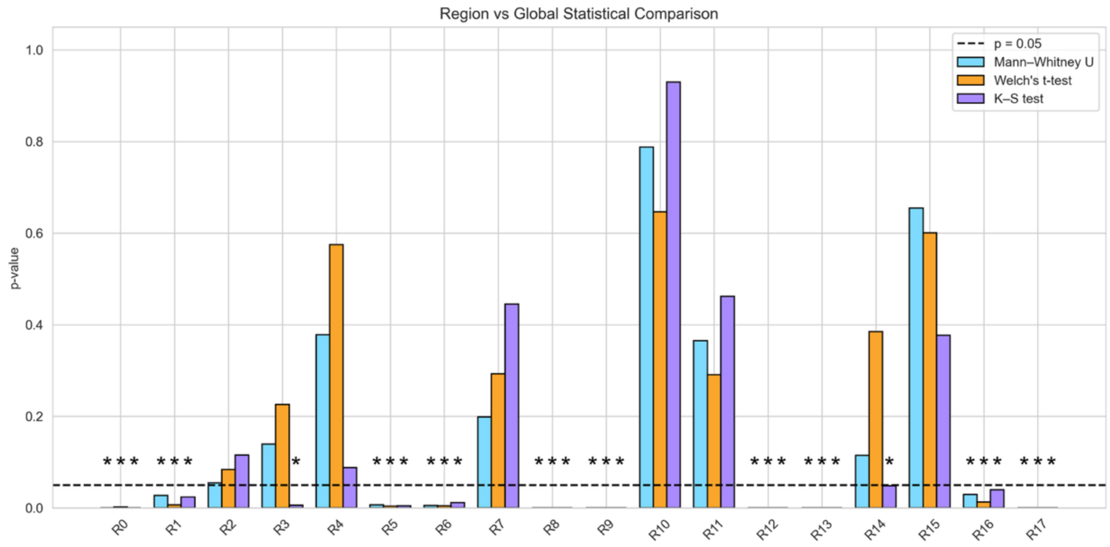

Barplot of p-values from statistical tests on region orientation data. p-values displayed for Mann-Whitney U-test (light blue), Welch's t-test (orange), K-S test (purple), per test regions. Horizontal dashed (--) line indicating an $\boldsymbol{\alpha}$ of $\mathbf{5}\%$ ($\boldsymbol{p - value = 0.05}$). Stars (*) used to indicate p-values below the threshold *p<0.05, **p<0.01, ***p<0.001.

Supplementary Table 1

P-values for statistical tests. Mann-Whitney U test and K-S test as displayed in Fig S2. Test ID is displayed on left column.

	p-value	
ID	Mann-Whitney U	KS
0	< 0.000	< 0.000
1	0.027	0.024
2	0.054	0.116
3	0.139	0.006
4	0.378	0.088
5	0.007	0.005
6	0.005	0.011
7	0.198	0.445
8	< 0.000	< 0.000
9	< 0.000	< 0.000
10	0.788	0.929
11	0.365	0.462
12	< 0.000	< 0.000
13	< 0.000	< 0.000
14	0.114	0.049
15	0.655	0.377
16	0.029	0.039
17	< 0.000	< 0.000

Supplementary Figure 3

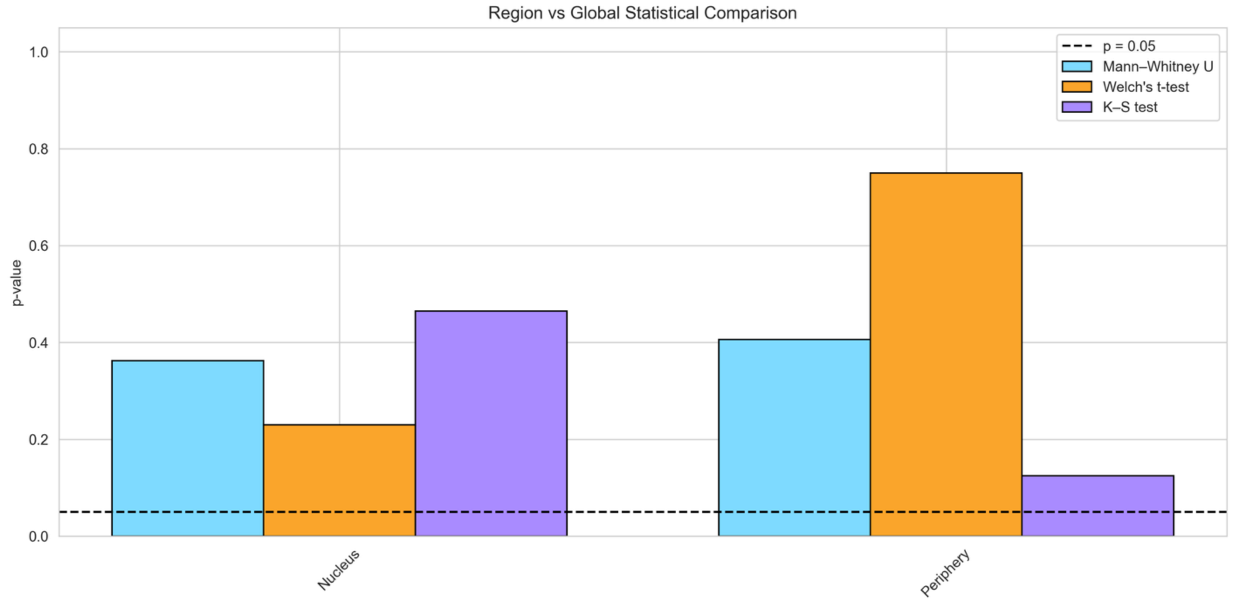

Barplot of p-values from statistical tests on cell landmark orientations. p-values displayed for Mann-Whitney U-test (light blue), Welch's t-test (orange), K-S test (purple), per test regions. Horizontal dashed (--) line indicating an $\boldsymbol{\alpha}$ of $\mathbf{5\%}$ ($\boldsymbol{p - value = 0.05}$). Stars (*) used to indicate p-values below the threshold.

Supplementary Figure 4

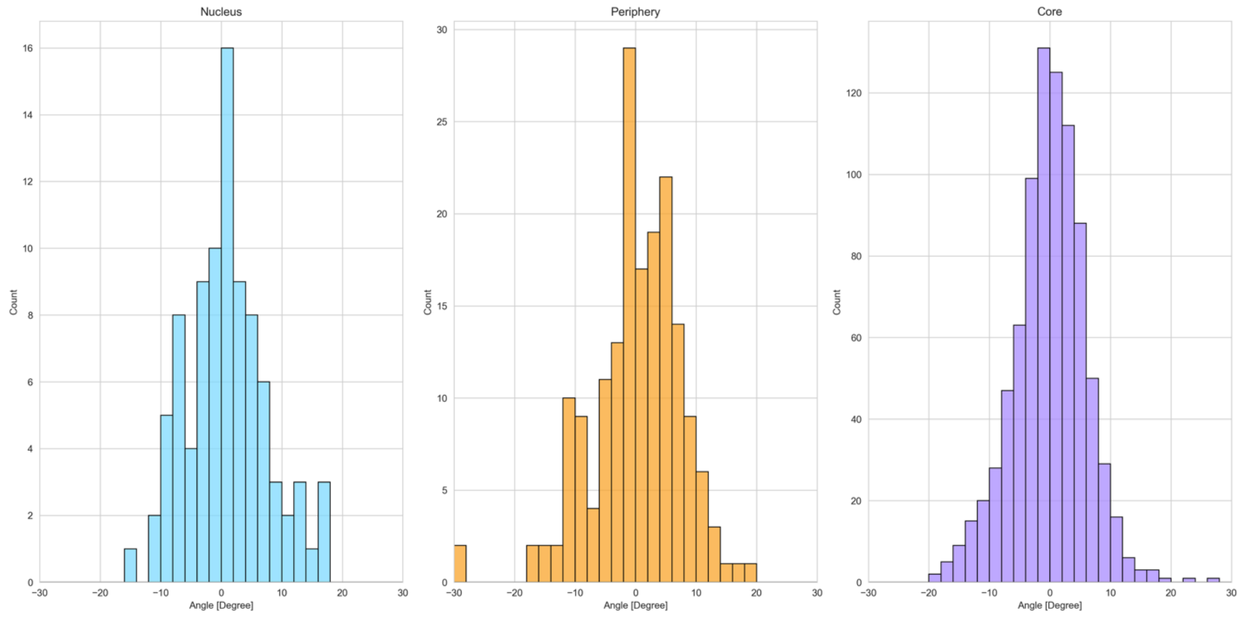

Histograms of landmarks orientation Z-Disc values. Distributions for nucleus (left; blue), periphery (middle, orange), and core (right, purple) regions. Domain is between $\mathbf{-30°}$ and $\mathbf{30°}$. Z-Discs directly adjacent to landmarks are included.

Supplementary Figure 5

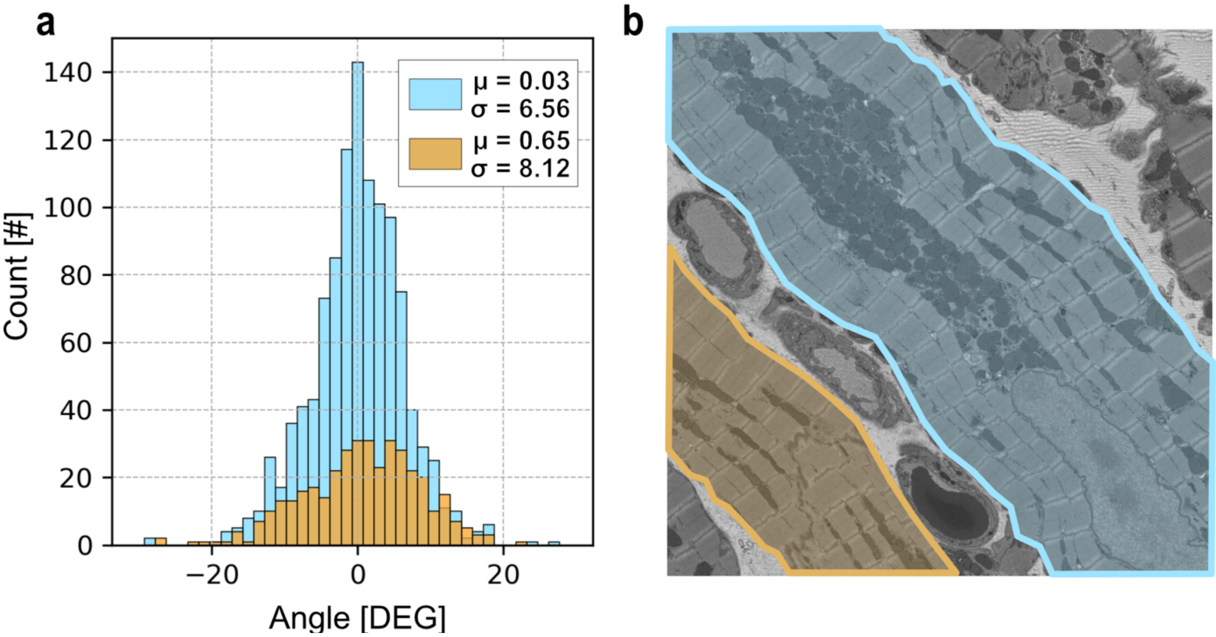

3D z-disc orientation of adjacent cardiomyocyte displays overlapping distribution. (a) Histogram of whole (main analysis) cardiomyocyte (blue, as seen in text, Fig. 3) and partial cardiomyocyte (orange) z-disc orientations overlapped. Orientation distributions overlap with similar means and standard deviations despite lower quantity of samples (whole, $\boldsymbol{n} = \mathbf{1121}$, partial, $\boldsymbol{n} = \mathbf{382}$). (b) 2D EM image of cardiomyocytes with highlights to indicate whole and partial cell.

Supplementary Table 2

Orientation profiles for two cardiomyocytes. The "director" is the eigenvector with which most orientation vectors align. Order (***S***) indicates how well the orientation vectors align with the director. Whole and partial cells align very strongly with the director which points in the contraction axis.

	Orientation profile	
	Whole	Partial
Director	$[-0.9998, \ 0.0194, \ -0.0071]$	$[-0.9998, \ 0.069, \ -0.020]$
Order (S)	0.9912	0.9752
Eigenvalues	$[0.9941, \ 0.0041, \ 0.0018]$	$[0.9835, \ 0.0134, \ 0.0032]$

Supplementary Table 3

Orientation Order (S) for isolated regions. Values demonstrate strong alignment across regions with major axis of the cell.

Order (S)								
R0	R1	R2	R3	R4	R5	R6	R7	R8
$\mathbf{0.9906}$	$\mathbf{0.9996}$	$\mathbf{0.9980}$	$\mathbf{0.9836}$	$\mathbf{0.9903}$	$\mathbf{0.9998}$	$\mathbf{0.9877}$	$\mathbf{0.9910}$	$\mathbf{0.9913}$
R9	R10	R11	R12	R13	R14	R15	R16	R17
$\mathbf{0.9982}$	$\mathbf{0.9956}$	$\mathbf{0.9504}$	$\mathbf{0.9983}$	$\mathbf{0.9985}$	$\mathbf{0.9958}$	$\mathbf{0.9910}$	$\mathbf{0.9988}$	$\mathbf{0.9823}$

Supplementary Figure 6

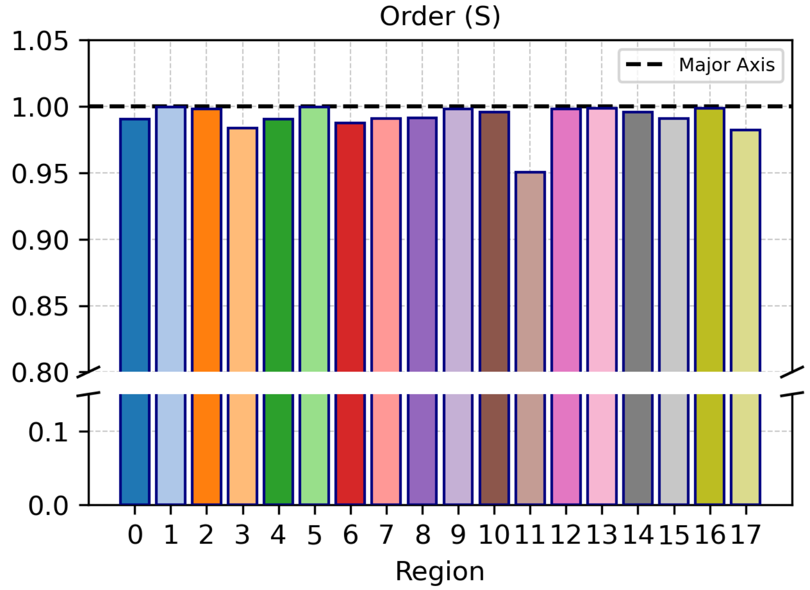

Alignment of each region with major axis demonstrated with Order (S). Bar-chart of order values (see Table S2) across regions shows strongly alignment for all with cell major axis. Region 11 has the least aligned z-discs. Break at 0.15 provided due to continuity of values.

Supplementary Figure 7

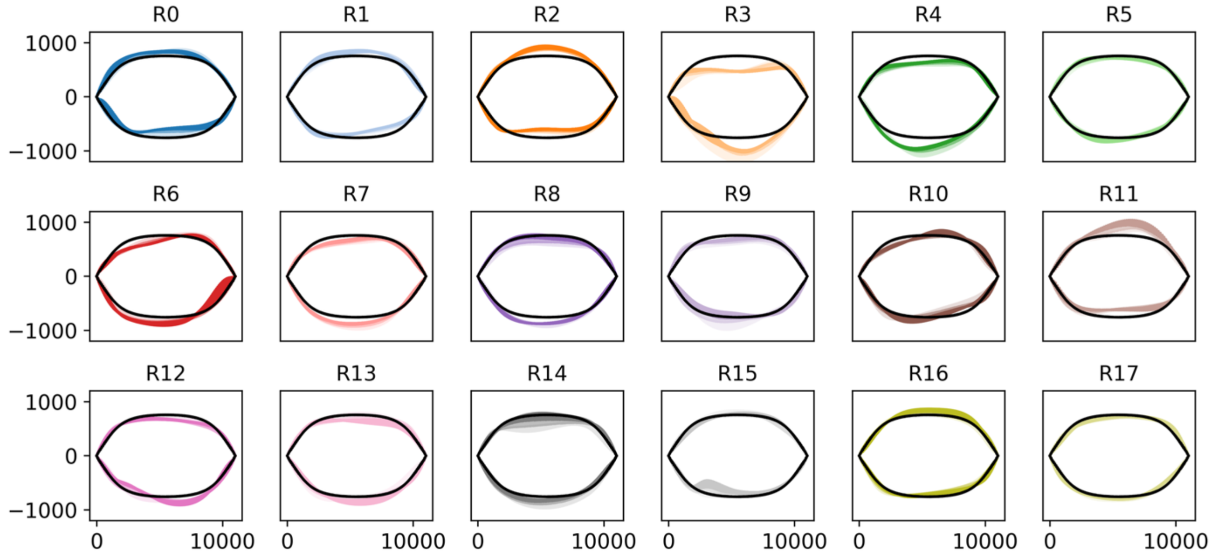

Y-Displacement of boundaries for each simulation overlayed on uniaxial case. [as in Fig. 5] Black solid lines are the boundary surface of the uniaxial simulation. Each coloured simulation is broken into three shaded components indicating thirds of the boundary depth.

Supplementary Figure 8

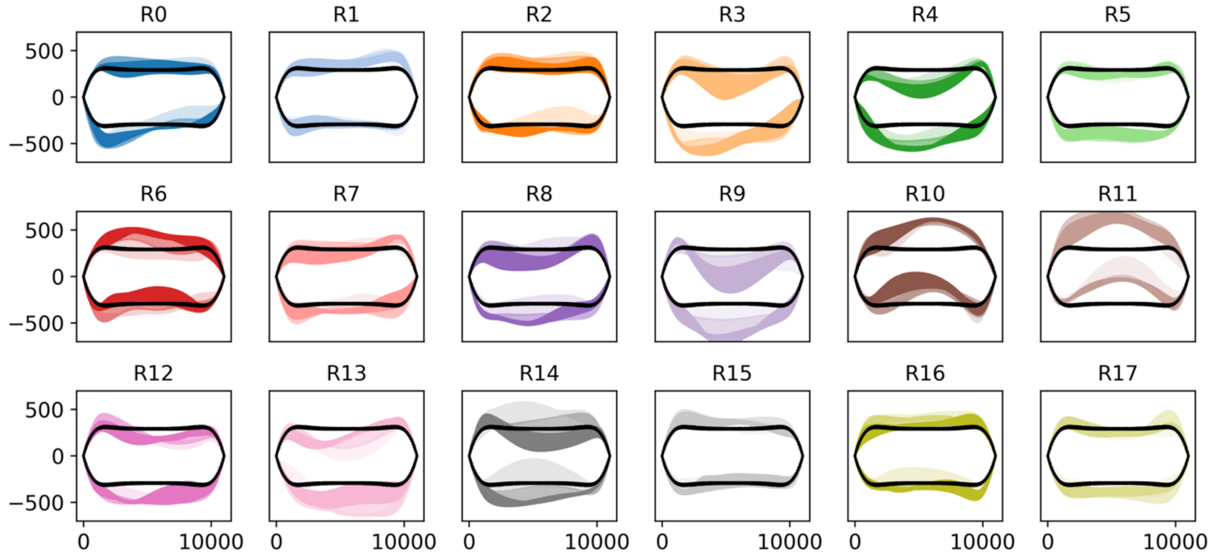

Z-Displacement of boundaries for each simulation overlayed on uniaxial case. [as in Fig. 5] Black solid lines are the boundary surface of the uniaxial simulation. Each coloured simulation is broken into three shaded components indicating thirds of the boundary depth.

Supplementary Figure 9

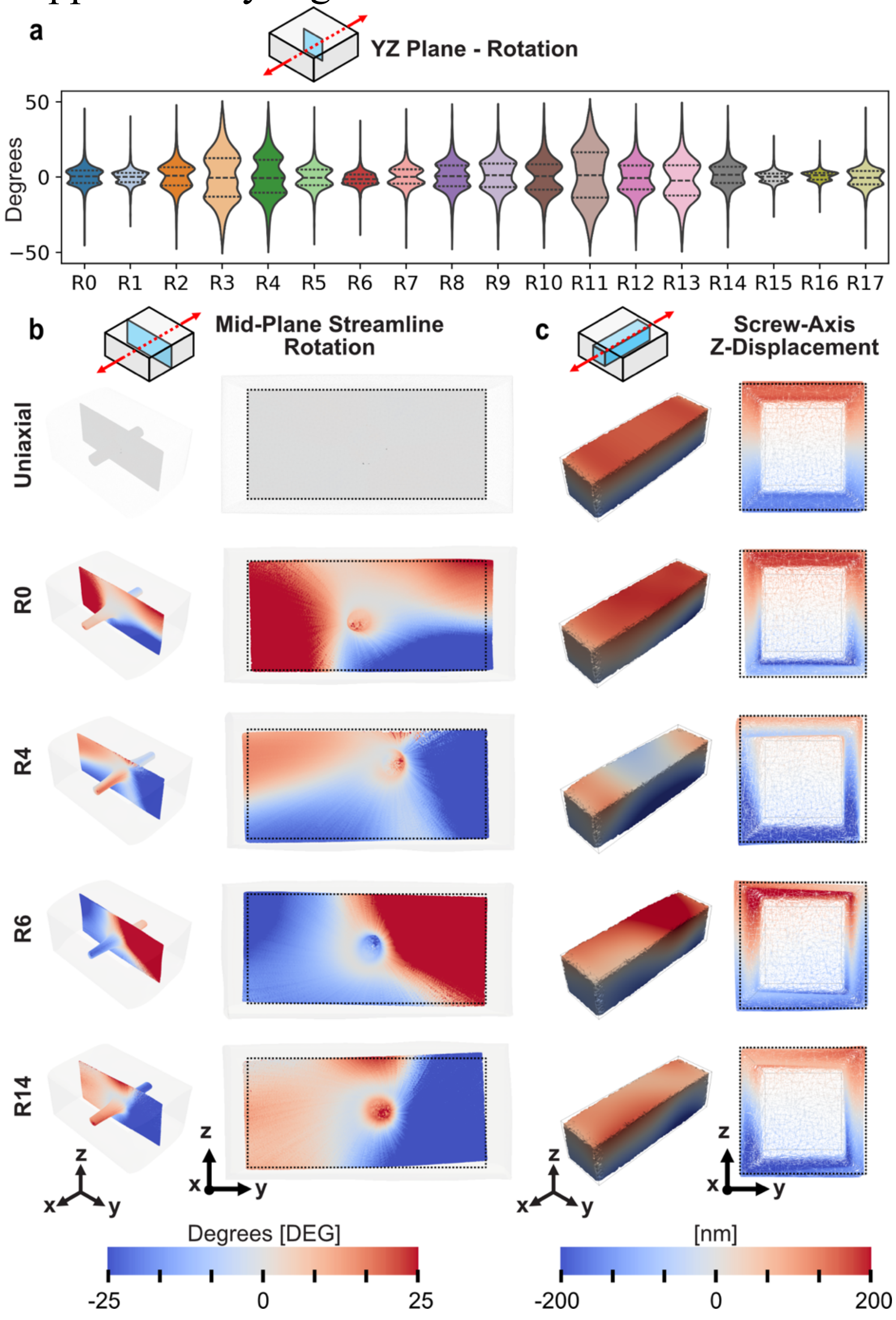

Streamline rotations in select simulations with internal displacement. (a) [as in Fig. 5] $\boldsymbol{yz}$-plane rotation caused by displacement displayed as violinplots with quartiles. Plane rotation determined at inner plane to reduce impacts of rigid boundaries. Violin plots show the distribution of common rotation values for each region, see Region 4 indicating frequent rotation values at $\approx \mathbf{-25°, 0, 25°}$. (b) Streamline plots of mid-plane rotation for select simulations and uniaxial case. Streamlines move from the centre of each test and merge in mid-plane. Streamline follows displacement gradient and is inevitably uniform for uniaxial contraction. (c) Displacements of internal beam about screw axis displaying unique behaviour in test cases due to internal rotation.

Supplementary Figure 10

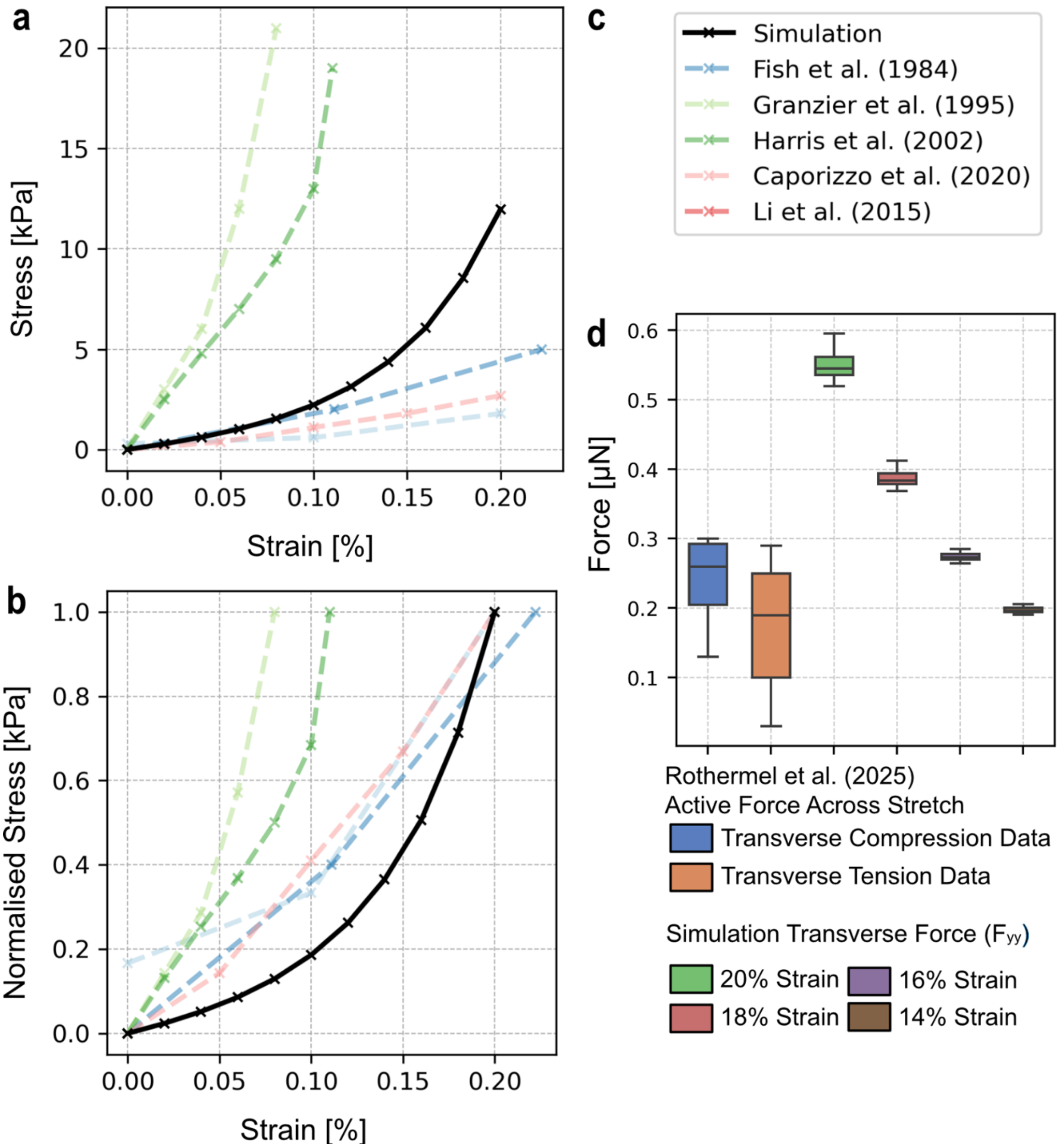

Tension curves and transverse peak values for experimental tests on cardiomyocytes versus simulation parameterisation. (a) Stress versus strain curves displaying characteristic exponential behaviour for tension alongside variety in calculated stress reported in literature. Simulation curve (black) parameterised to balance reported results. (b) Stress normalised by peak tension versus strain demonstrating agreements in trend structure. (c) Key for plots (a) and (b). (d) Boxplot of transverse active force production for Compression and Tension stretch, Rothermel et al. (2025), compared to simulation forces. Experiment boxplots demonstrate values for multiple strain levels. Simulation transverse forces for **20**%, **18**%, **16%,** and **14**% compression showing agreement in range.

Supplementary Figure 11

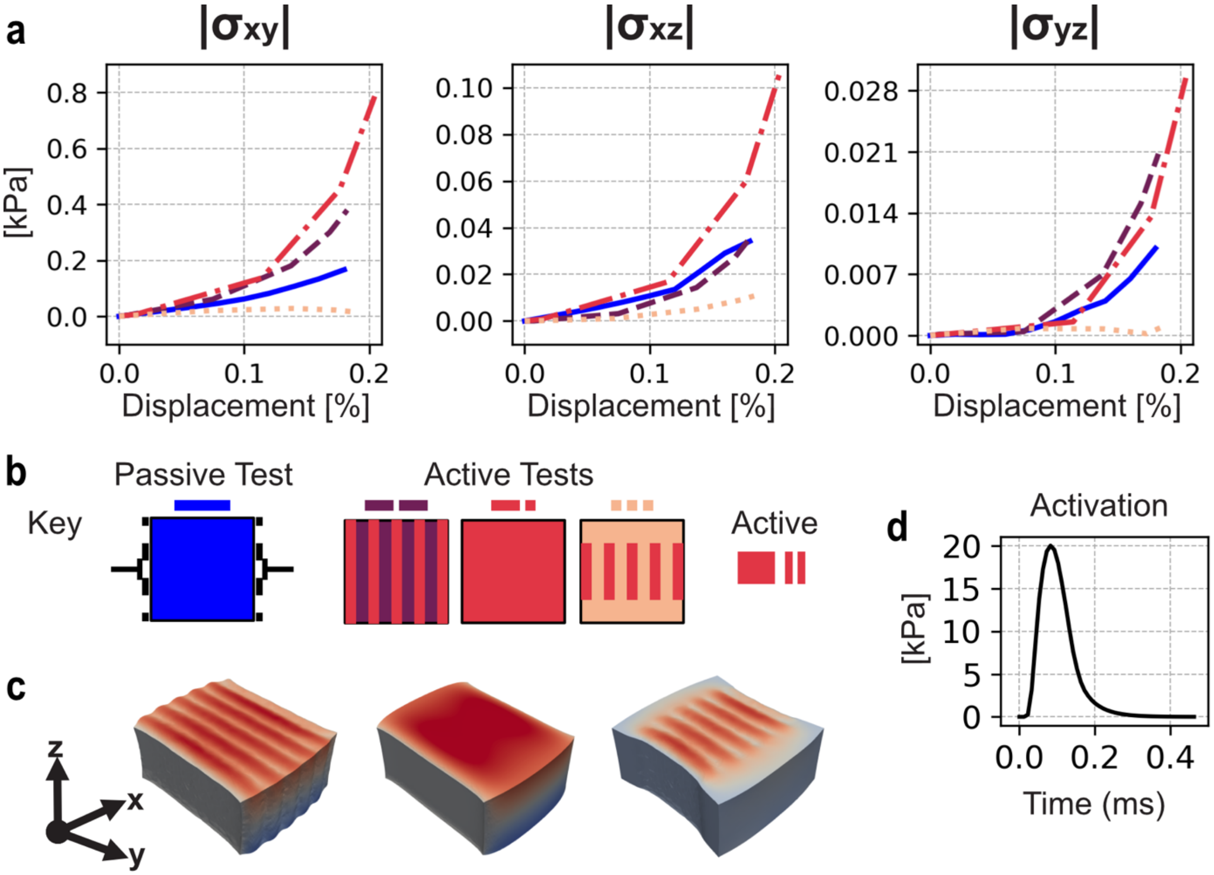

Shear stress behaviour compared between passive simulation with discrete boundary conditions and active tests. (a) Magnitude of shear stresses ($\boldsymbol{\sigma_{xy}}$, $\boldsymbol{\sigma_{xz}}$, $\boldsymbol{\sigma_{yz}}$) over full displacement window. Passive simulation (solid line, blue) as shown in main results (not small y-axis). Active contraction tests, where active stress is applied in periodic sarcomeres across whole width (dashed [--] line, purple), all nodes within volume (dash dot [-.] line, red), and in periodic sarcomeres across middle (dotted [..], pink). See (b) for Key indicating active stress patterns. (c) Renders of active contraction tests at peak stress with z-displacement shown for visualisation purposes. (d) Active stress curve.

Supplementary Table 4

Comparison of Christoffel Symbol and Stress across simulations. Magnitude of Christoffel Symbol shown to demonstrate collapse during uniaxial testcase. Maximum stress during 0 displacement remains null for all tests.

Test	Magnitude of Christoffel Symbol	Maximum Stress at 0 Load [kPa]
Uniaxial	9.16×10^{-18}	0.00
R0	1.18×10^{-4}	0.00
R4	3.99×10^{-5}	0.00
R6	5.96×10^{-5}	0.00
R14	6.79×10^{-5}	0.00

Supplementary Figure 12

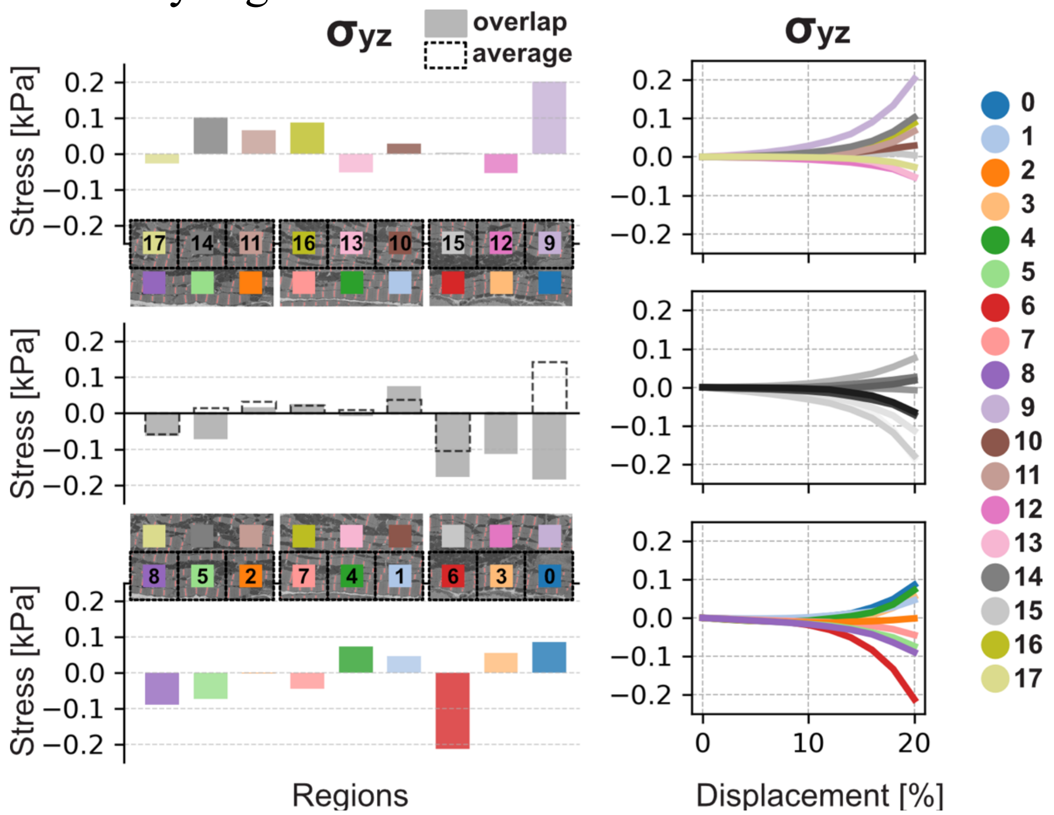

Trends for *yz*-shear stress and z-disc orientation. (left) Bar charts and (right) trends of shear stress (yz) for each region. (top) Upper region ($\boldsymbol{y > 9.9}$ [$\boldsymbol{nm}$]) shear shows positive behaviour in both final value bar charts and trends. (middle) Overlap region simulations (solid) and average between upper and lower simulations (dashed, [--]).
